# PINX1 loss confers susceptibility to PARP inhibition in pan-cancer cells

**DOI:** 10.1038/s41419-024-07009-6

**Published:** 2024-08-22

**Authors:** Mei Huang, Xiaotong Zhu, Chen Wang, Liying He, Lei Li, Haopeng Wang, Gaofeng Fan, Yu Wang

**Affiliations:** 1 School of Life Science and Technology, ShanghaiTech University, Shanghai, China; 2 Shanghai Clinical Research and Trial Center, Shanghai, 201210 China; 3 Department of Gynecology, Shanghai First Maternity and Infant Hospital, School of Medicine, Tongji University, Shanghai, 200092 China; 4 Shanghai Key Laboratory of Maternal Fetal Medicine, Shanghai Institute of Maternal-Fetal Medicine and Gynecologic Oncology, Clinical and Translational Research Center, Shanghai First Maternity and Infant Hospital, School of Medicine, Tongji University, Shanghai, China

**Keywords:** Oncogenes, Cell biology, DNA damage and repair, Transcription

## Abstract

PARP1 is crucial in DNA damage repair, chromatin remodeling, and transcriptional regulation. The principle of synthetic lethality has effectively guided the application of PARP inhibitors in treating tumors carrying BRCA1/2 mutations. Meanwhile, PARP inhibitors have exhibited efficacy in BRCA-proficient patients, further highlighting the necessity for a deeper understanding of PARP1 function and its inhibition in cancer therapy. Here, we unveil PIN2/TRF1-interacting telomerase inhibitor 1 (PINX1) as an uncharacterized PARP1-interacting protein that synergizes with PARP inhibitors upon its depletion across various cancer cell lines. Loss of PINX1 compromises DNA damage repair capacity upon etoposide treatment. The vulnerability of PINX1-deficient cells to etoposide and PARP inhibitors could be effectively restored by introducing either a full-length or a mutant form of PINX1 lacking telomerase inhibitory activity. Mechanistically, PINX1 is recruited to DNA lesions through binding to the ZnF3-BRCT domain of PARP1, facilitating the downstream recruitment of the DNA repair factor XRCC1. In the absence of DNA damage, PINX1 constitutively binds to PARP1, promoting PARP1-chromatin association and transcription of specific DNA damage repair proteins, including XRCC1, and transcriptional regulators, including GLIS3. Collectively, our findings identify PINX1 as a multifaceted partner of PARP1, crucial for safeguarding cells against genotoxic stress and emerging as a potential candidate for targeted tumor therapy.

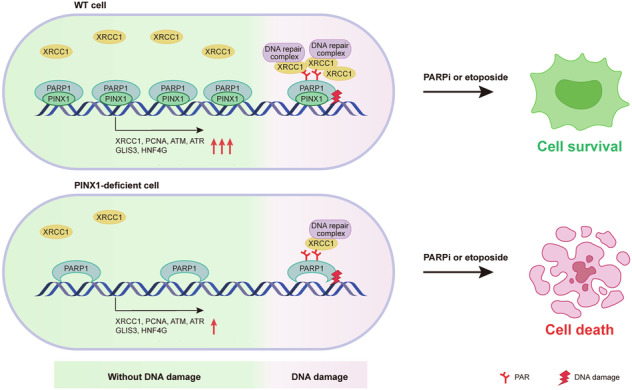

## Introduction

PARP1, an extensively studied target for cancer therapy, plays a pivotal role in the DNA damage response by covalently transferring ADP-ribose from NAD^+^ to target substrates, producing poly (ADP-ribose) (PAR) polymers. PARP1 consists of three main functional domains: a DNA binding domain composed of three zinc fingers (ZnF1, ZnF2, and ZnF3) at the N-terminal, a central auto-modification domain with a BRCT module, and a highly conserved C-terminal catalytic domain [1]. As an early and upstream sensor of diverse DNA lesions, PARP1 is recruited to DNA damage sites within seconds, undergoing rapid activation to catalyze PARylation of substrates, including histones and itself [2]. This PARylation process serves dual functions in DNA damage response. Firstly, the charge repulsion of highly negatively charged PAR chains promotes chromatin decondensation. Secondly, the PAR chains directly mediate the recruitment of DNA damage repair machinery, including XRCC1 [1, 3]. Additionally, the excessive auto-modification of PARP1 aids in its dissociation from DNA lesions, facilitating subsequent repair processes [1, 4].

While PARP1 is known to be the primary responder in single-strand break (SSB) repair, homologous recombination (HR) dependent on BRCA is a critical mechanism for precise double-strand break (DSB) repair in cells. Exploiting the concept of synthetic lethality, PARP inhibitors (PARPis) have proven effective in treating HR-deficient cancers, particularly those lacking functional BRCA1 or BRCA2 [5]. However, a growing body of evidence suggests that PARPis also confer significant benefits for some HR-proficient cancer patients [5–8], indicating the existence of other potential biomarkers and mechanisms contributing to the cytotoxicity of PARPis in cancer cells.

Initially, it was believed that PARPis killed cancer cells by inhibiting the catalytic activity of PARP1. However, subsequent discoveries revealed that the “trapping” ability of PARPis, hindering the dissociation of PARP1 from DNA lesions, also contributed to their lethality [9–11]. Interestingly, in addition to the synthetic lethality resulting from BRCA deficiency, blocking or deleting some PARP1 interactors has also synergized with PARP inhibitors. This synergy arises from the interactors’ direct regulatory effect on PARP1, with some (including XRCC1 and ALC1) modulating the dissociation of PARP1 from DNA lesions [12–15], and others (including HPF1 and PNUTS) regulating the enzymatic activity of PARP1 [16–18]. These interactions ultimately impact the sensitivity and efficacy of PARP inhibitors. Furthermore, recent studies have unveiled the diverse roles of PARP1 in various cellular processes beyond DNA damage repair, including transcriptional regulation, chromatin remodeling, and RNA processing [19, 20]. These findings open up additional therapeutic opportunities for PARP inhibitors.

In this context, we aimed to broaden our comprehension of the biological roles of PARP1 and the applicability of PARP inhibitors by exploring novel PARP1 interacting proteins. Utilizing TurboID, an efficient and non-toxic proximity labeling method [21], we mapped unknown partners of PARP1. Our investigation unveiled three interactors—PINX1, UTP14A, and ZNF24—that were not previously reported to be associated with PARP1. Notably, the deletion of PINX1 exhibited a synergistic effect with PARP inhibition in cancer cells.

PINX1 (PIN2/TRF1-interacting telomerase inhibitor 1) was initially identified as an interacting protein of TRF1 in the telomeric shelterin complex and recognized as a potent intrinsic telomerase inhibitor, hence considered a tumor suppressor [22, 23]. However, studies have also demonstrated PINX1’s involvement in promoting tumor progression in various cancers [24–28], raising questions about its exclusive role as a telomerase inhibitor or tumor suppressor. Our study revealed that PINX1 maintains cellular DNA damage repair capacity independently of telomerase inhibition, introducing a new facet to PINX1’s function as a partner of PARP1, whose deletion sensitizes tumor cells to PARPi.

## Material and methods

### Cell culture

HEK293T (ATCC CRL-3216), Hela (ATCC CCL-2), U2OS, OVCAR8, OC316, and BEL7404, authenticated by ATCC, were used for cell-based experiments. Mycoplasma contamination was examined by PCR. HEK293T, Hela, and U2OS cells were cultured in Dulbecco’s modified Eagle’s medium (DMEM) supplemented with 10% fetal bovine serum (FBS), penicillin (100 units/ml), and streptomycin (100 μg/ml), and maintained at 37 °C in 5% CO_2_. OVCAR8, OC316, and BEL7404 cells were grown in Roswell Park Memorial Institute (RPMI) 1640 medium supplemented with 10% FBS and Pen/Strep and grown in the same conditions noted above. U2OS was kindly provided by Prof Lei Li’s laboratory at ShanghaiTech University. OVCAR8 was obtained from Ahmed Ashour Ahmed’s lab in the Ovarian Cancer Cell Laboratory, University of Oxford. BEL7404 was a gift from Prof. Yong Cang’s laboratory at ShanghaiTech University.

### Cell transfection and infection

We followed the manufacturer’s protocol of Mirus (TransIT-2020, Mirus Bio) to perform transient transfection. Briefly, cells were plated in a 6-well plate or a 6-cm dish 24 h before transfection. When cells reached ~75% confluence, we prepared Mirus: plasmid complexes in Opti-MEM I Reduced Serum Medium (Gibco) and added them into each well.

Cell lines with gene-stable expression were established by lentiviral infection, followed by fluorescence-activated cell sorting or puromycin selection. In brief, lentivirus was generated in HEK293T cells by co-transfecting the gene-containing lentiviral construct, psPAX2, and pMD2.G at a ratio of 3:2:1; 48–72 h later, supernatants were collected and passed through 0.45 μm filters to remove cell debris. The viral supernatant was then added to cells to be infected. Infected cells were either sorted by fluorescence or selected by puromycin. The effectiveness of infection was confirmed by flow cytometry or western blot.

### TurboID and mass spectrometry analysis

Cells were transiently transfected with a construct expressing full-length PARP1 tagged on the C-terminus with TurboID or a construct expressing TurboID alone. Three biological replicates were prepared for each condition. After 24 h, cells were treated with DMSO or 500 μM biotin for 10 min at 37 °C before harvesting by trypsinization. Cells were washed five times with ice-cold PBS and lysed with lysis buffer (50 mM Tris-HCl pH 7.5, 200 mM NaCl, 2% Triton X-100, 0.1% SDS, and 1× protease inhibitor cocktail) on ice for 15 min. Cell lysates were centrifuged at 15,000 × *g* for 15 min at 4 °C, and supernatants were collected. The powdered form of urea was added to a final concentration of 8 M and DTT to a final concentration of 10 mM. Then, the lysates were incubated at 56 °C for 1 h with shaking. Iodoacetamide was added to a final concentration of 25 mM. Samples were incubated in the dark for 45 min at room temperature. The alkylation was stopped by adding DTT to a final concentration of 25 mM and setting it at room temperature for 30 min. The samples and magnetic streptavidin beads were incubated at 4 °C overnight. The beads were washed twice in sequence with the following buffers: buffer 1 (50 mM Tris-HCl pH 8.0, 200 mM NaCl, 8 M urea, 0.2% SDS), buffer 2 (50 mM Tris-HCl pH 8.0, 200 mM NaCl, 8 M urea), buffer 3 (50 mM Tris-HCl pH 8.0, 0.5 mM EDTA, 1 mM DTT). Biotinylated proteins were then eluted from the beads by boiling the beads in 3× protein loading buffer (6% SDS, 15% glycerol, 180 mM Tris-HCl pH 6.8, 3.6% 2-Hydroxy-1-ethanethiol, 0.12% bromophenol blue) supplemented with 20 mM DTT and 2 mM biotin. The sample preparation for mass spectrometry analysis was conducted as previously described [29].

### Immunoprecipitation (IP) and western blot analysis

Cultured cells were lysed with Triton X-100 lysis buffer (50 mM Tris-HCl pH 8.0, 100 mM NaCl, 1% Triton X-100) supplemented with 5 mM MgCl_2_, 1× protease inhibitor cocktail, 1 μM Olaparib (Selleck, Cat# S1060), 1 μM PARGi PDD00017273 (APExBIO, Cat# B8394) at 4 °C. Cell lysates were centrifuged at 15,000 × *g*, and supernatants were collected. For the IP of endogenous proteins, supernatants/purified proteins were subjected to immunoprecipitation with corresponding antibodies. After incubation at 4 °C overnight, protein A/G beads (GE) were added and incubated for another 4 hours. Anti-tag beads were directly added to supernatants/purified proteins for the IP of ectopic expressed proteins with tags and incubated at 4 °C overnight. Immunoprecipitates were washed 3 times with the Triton X-100 lysis buffer, followed by the addition of 5× protein loading buffer (10% SDS, 25% glycerol, 300 mM Tris-HCl pH 6.8, 6% 2-Hydroxy-1-ethanethiol, 0.2% bromophenol blue). Samples were subjected to western blot analysis. Membranes were blocked in 5% milk in TBST (TBS/Tween 20: 20 mM Tris HCl, pH 7.5, 50 mM NaCl, and 0.1% Tween 20) for 1 hour at room temperature on a shaker and incubated with primary antibody at 4 °C overnight. Proteins were detected with horseradish peroxidase (HRP)-conjugated secondary antibodies (Jackson Laboratory) and ECL (PerkinElmer, Cat# NEL105001EA).

Following primary antibodies were used for western blot analysis in this study: FLAG (GNI, Cat# GNI4110-FG), Myc (Cell Signaling, Cat# 2276S), GAPDH (Proteintech, Cat# 10494-1-AP-100UL), γ-tubulin (Sigma-Aldrich, Cat# T6557), Vinculin (Sigma, Cat# V4505), PINX1 (Proteintech, Cat# 12368-1-AP-50ul), PAR (R&D, Cat# 4335-MC-100), PARP1 (Cell Signaling, Cat# 9542S), Lamin B1 (Bimake, Cat# A5106), β-tubulin (Eastacres Biologicals, Cat# MaTub-b), XRCC1 (Bimake, Cat# A5299), PCNA (Santa Cruz, Cat# sc-7907), γH2AX (Millipore, Cat# 05-636), Histone H3 (Bimake, Cat# A5885), Lamin B (Santa Cruz, Cat# sc-6216), FLAG (Abclonal, Cat# AE063), Myc (Abclonal, Cat# AE070), UTP14A (Abclonal, Cat# A5960), ZNF24 (Santa Cruz, Cat# sc-393359), ATM (Beyotime, Cat# AF1399), ATM p-Ser1981 (Abclonal, Cat# AP0008), ATR (Beyotime, Cat# AF6267), ATR p-Thr1989 (GeneTex, Cat# GTX637560-S), GFP (BBI, Cat# D110008), mCherry (OriGene, Cat# AB8181-200).

### shRNA-based gene knockdown

All the sequences of designed shRNAs were listed in Supplementary Table S1. The plasmid pLKO.1 (Addgene, Cat# 10878) was used as the shRNA construct backbone. Virus packaging and infection were performed as described above. The infected cells were selected with 1.5–5 µg/ml puromycin for at least 5 days.

### CRISPR/Cas9-based gene knockout

To generate PARP1 or PINX1 KO cell lines using the CRISPR-Cas9 system, the CRISPR sgRNA database (http://crispr.mit.edu) was applied to generate sgRNAs for each gene. All the sequences of designed sgRNAs were listed in Supplementary Table S1. The selected sgRNAs were then subcloned into PX330–Cas9–GFP plasmid, followed by transient transfection into cells and FACS for GFP-positive single clone. The KO effect was confirmed by western blot analysis with relevant antibodies.

### γH2AX immunofluorescence staining and imaging

Cells were grown on coverslips in 24-well plates and treated as indicated. Cells were washed once with PBS and fixed with 10% Neutral Formalin Fix Solution (BBI, Cat# E672001) for 15 min at room temperature and washed three times with PBS. The cells were then permeabilized with 0.5% Triton X-100 in PBS for 10 min and blocked with 2% BSA in Cell Staining Buffer (Biotech, Cat# FXP005) for 60 min. Fixed cells were incubated with a γH2AX antibody (1:500; Millipore, Cat# 05-636) at 4 °C overnight, washed three times with PBS, and incubated with an Alexa Fluor 647-conjugated anti-mouse secondary antibody (1:1000; Thermo Fisher Scientific, Cat# A21235) at dark for 1 h at room temperature. Cells were then stained with 1 μg/ml DAPI for 10 min and washed three times with PBS for 5 min. Coverslips were mounted onto slides with a mounting medium. Fluorescent images were captured with a Nikon CSU-W1 Sora Spinning Disk confocal microscope using a 60×/1.4 oil objective and analyzed using ImageJ software.

### Laser micro-irradiation and imaging

Cells were plated in a 35-mm glass bottom dish with a 0.17-mm coverslip bottom (cellvis, Cat# D35-20-1.5-N). Twelve to twenty-four hours after seeding, EGFP or mCherry fused protein constructs and indicated other constructs were transfected into cells. Twenty-four hours later, the transfected cells were pre-sensitized with 10 μg/ml Hoechst 33342 at 37 °C for 10 min before irradiation. Cells were imaged with a Nikon CSU-W1 Sora Spinning Disk confocal microscope using a 60×/1.4 oil objective. The region of interest in each nucleus was selected for cells with indicated protein expression. The micro-irradiation was generated by a 405 nm laser set to 50% transmission to scan the selected ROI for 100 ms to induce DNA damage.

For immunostaining, cells following irradiation were fixed with 10% neutral formalin fix solution for 15 min at room temperature and washed three times with PBS. The cells were then permeabilized with 0.5% Triton X-100 in PBS for 10 min and blocked with 2% BSA in cell staining buffer for 60 min. Fixed cells were incubated with an anti-FLAG Monoclonal antibody (1:500; GNI, Cat# GNI4110-FG), γH2AX antibody (1:500; Millipore, Cat# 05-636), or p-ATM antibody (1:300; Abclonal, Cat# AP0008) at 4 °C overnight, washed three times with PBS, and incubated with an Alexa Fluor 647-conjugated anti-mouse secondary antibody (1:1000; Thermo Fisher Scientific, Cat# A21235) or an Alexa Fluor 488-conjugated anti-rabbit secondary antibody (1:1000; Beyotime, Cat# A0423) in the dark for 1 h at room temperature. Coverslips were mounted onto slides with a mounting medium. Fluorescent images were captured by Zeiss LSM 800 Confocal microscope or Nikon CSU-W1 Sora Spinning Disk confocal microscope, and processed using ImageJ software. Colocalization analysis was performed with Coloc2 in ImageJ. ROIs, including the irradiated region within the nucleus were selected, and Pearson’s *R* value was calculated between two channels without threshold.

Time-lapse images were captured pre- and post-irradiation for live cell imaging as indicated. In each experiment, at least 36 cells were irradiated in 3 sets of 12, and the experiments were performed at least two times on different days. For the quantitative evaluation of the recruitment kinetics, mean fluorescence intensities of the irradiated regions were measured over time by NIS Element High Content Analysis software (Nikon Inc.), collecting fluorescence intensities of blank regions as background. Relative recruitment was defined as the ratio of the corrected mean fluorescence intensity in and outside micro-irradiated areas, which makes the relative recruitment before irradiation 1 and means that relative recruitment >1 represents an enrichment of the protein at the irradiated sites.

### Alkaline comet assay

Cells were treated with 10 µM etoposide for 30 minutes and recovered for 30 min. Alkaline comet assay was performed as described previously [30]. In brief, 0.5% low melting point agarose (Collins, Cat# X2802361) was prepared at 42 °C to mix with cells (about 20,000 cells in 100 μl low melting point agarose), spread on frosted slides, and set at 4 °C for 15 min, and then lysed in alkaline lysis buffer (2.5 M NaCl, 100 mM EDTA, 10 mM Tris-base, 1% Triton X-100, 1% DMSO, pH 10.0) at 4 °C for 1 h followed by washing for three times. Samples were placed into electrophoresis buffer (300 mM NaOH, 1 mM EDTA) for 20 min, and then electrophoresis was performed in an ice bath for 20 min (approx. 0.8–1.5 V/cm). After electrophoresis, the samples were neutralized with neuralization buffer (0.4 M Tris-Cl, pH 7.5) 3 times. Slides were drained and stained with SYBR Green I (Medchemexpress, Cat# HY-K1004) for 20 min. The images were captured by a fluorescence microscope and analyzed using the OpenComet plugin installed in ImageJ software (https://cometbio.org).

### Cell survival assay

Two hundred cells per well for Hela, 500 cells per well for OVCAR8 and OC316, and 600 cells per well for BEL7404 were seeded in white 96-well plates and treated with indicated drugs at different concentrations 18–24 h after initial seeding. CellTiter-Glo^®^ Luminescent Cell Viability Assay (CTG) (Promega, Cat# G7572) was used to evaluate the survival at day 6 after treatment, according to the manufacturer’s instructions. The luminescence readout was performed on SpectraMax i3 (MD).

### Animal work

Mice were raised in the animal facility of the National Facility for Protein Science in Shanghai (NFPS). The animal house is temperature-controlled with a 12-hour light/dark cycle. Food and water were available ad libitum. All study protocols involving mice were approved by the Institutional Animal Care and Use Committee of ShanghaiTech University and conducted under governmental regulations of China for the care and use of animals. Six to eight-week-old NSG mice were used.

Xenograft tumors were established by subcutaneously injecting 2 × 10^6^ OC316 shLuc or shPINX1 (PINX1 sh2) cells suspended in 100 μl PBS (Gibco, Cat# C20012500BT). When tumor volume reached approximately 200 mm^3^, the mice were selected based on tumor volume and randomly assigned to treatment and vehicle control groups with 5 animals in each group. Mice in the treatment groups were administered by oral gavage once a day with 0.33 mg/kg Talazoparib (Selleck, Cat# S7048) formulated in 5% (v/v) DMSO and 95% (v/v) ddH_2_O. The control group mice were treated with equal volumes of vehicle daily via oral gavage. Tumor size was measured using electronic calipers periodically during treatment. The maximal tumor burden permitted by our ethics committee is no more than 2000 mm^3^. Before the tumor burden reached 2000 mm^3^, mice were euthanized, and the tumors were dissected for further analysis. All the tumors were removed, photographed, and weighed.

### Chromatin fractionation

Chromatin fractionation was carried out according to the procedure of Blessing et al. [31] with some modifications [31]. In brief, 1.5 × 10^6^ cells were seeded in 6 cm dishes. Twenty-four hours after seeding, cells were treated with indicated drugs. After treatment, cells were digested with a 0.25% trypsin–EDTA solution, collected by centrifugation at 500 × *g* for 3 min, and washed once with ice-cold PBS. Cells were subsequently pelleted and resuspended in 4× the pellet volume lysis buffer (30 mM Tris-HCl pH 7.5, 150 mM NaCl, 0.5% Triton X-100, 2 mM MgCl_2_ and 1× Protease Inhibitor Cocktail). 1/10 volume of suspension was directly added to 5× protein loading buffer as whole cell lysate (WCL). The rest of the suspensions were incubated on ice for 15 min and centrifuged for 15 min at 15,000 × *g* at 4 °C to pellet the chromatin. The supernatant was used as a soluble fraction. The pellet was washed three times with lysis buffer. The sample was vortexed for each washing step, and chromatin was pelleted for 5 min at 15,000 × *g* at 4 °C. The chromatin pellet was dissolved in a solution containing 8 mM NaOH and 1% SDS. All samples were boiled at 95 °C for 15 min after adding 5× protein loading buffer. Samples were centrifuged for 15 min at 15,000 × *g* to collect the supernatant and subjected to western blot analysis.

### Quantitative real-time PCR for mRNA

Total RNA was extracted using the Trizol reagent (Beyotime, Cat# R0016) according to the manufacturer’s instructions. RNA was used for cDNA synthesis with HiScript II Q RT SuperMix for qPCR (Vazyme, Cat# R223). Quantitative Real-Time PCR was performed using ChamQ Universal SYBR qPCR Master Mix (Vazyme, Cat# Q711) on the Quantstudio 7 system. The expression of each gene was calculated relative to the expression of GAPDH and represented the mean ± SD of three independent experiments.

### Chromatin immunoprecipitation sequencing (ChIP-seq)

Two biological replicates were prepared for each condition. Cells were grown to ~80–90% confluence, cross-linked with 1% paraformaldehyde in PBS for 10 minutes at room temperature, and quenched in 125 mM glycine in PBS for 5 min. Cells were washed twice with ice-cold PBS and collected by centrifugation at 5000 × *g* for 5 min, then sonicated in nuclei lysis buffer (50 mM Tris-HCl, pH 8.0, 10 mM EDTA, 1% SDS, 1× protease inhibitor cocktail) with Bioruptor Plus (Diagenode) to generate chromatin fragments of ~500 bp in length. The material was clarified by centrifugation at 20,000 × *g* for 10 min at 4 °C and diluted 10-fold in dilution buffer (1.1% Triton X-100, 1.2 mM EDTA, 167 mM NaCl, 16.7 mM Tris-HCl pH 7.9, 1× protease inhibitor cocktail), and pre-cleared with protein A/G agarose beads at 4 °C for 1 h. The pre-cleared, chromatin-containing supernatant was incubated with anti-PARP1 antibody (Cell Signaling, Cat# 9532) or rabbit IgG (Cell Signaling, Cat# 2729) as a control at 4 °C overnight. Protein A/G beads were added, incubated at 4 °C for 1 h, and washed once in sequence with the following buffers: low salt buffer (20 mM Tris-HCl pH 8.0, 2 mM EDTA, 0.1% SDS, 1% Triton X-100, 150 mM NaCl), high salt buffer (20 mM Tris-HCl pH 8.0, 2 mM EDTA, 0.1% SDS, 1% Triton X-100, 500 mM NaCl), LiCl buffer (0.25 M LiCl, 1% NP-40, 1% sodium deoxycholate, 1 mM EDTA, 10 mM Tris-HCl pH 8.0) and finally washed twice with TE buffer (10 mM Tris-HCl pH 8.0, 1 mM EDTA). The immunoprecipitated protein-DNA complex was eluted with elution buffer (1% SDS, 0.1 M NaHCO_3_), and cross-linking was reversed by adjusting to 200 mM NaCl and incubating for 6 h at 65 °C. Residual RNA and protein were cleared by digestion with RNase and proteinase K, respectively. ChIP-enriched and corresponding input DNA was purified with a MinElute PCR Purification Kit (QIAGEN). GENEWIZ from Azenta Life Sciences (China) conducted library construction, sequencing, and raw data processing.

### Quantification and statistical analysis

Data were analyzed using GraphPad Prism 8 software, with students’ *t*-tests and two-way ANOVA employed as appropriate. Figure legends described the number of biological replicates (*n*), and means ± SDs or SEMs used for error bars were also indicated in the legends. The following indications of significance were used throughout the paper and displayed for each experiment in the figure legends: *, *p* < 0.05; **, *p* < 0.01; ***, *p* < 0.001; ****, *p* < 0.0001; and ns indicated no significance (*p* > 0.05).

## Results

### Identification of novel binding proteins for PARP1 by TurboID

**Fig. 1:**
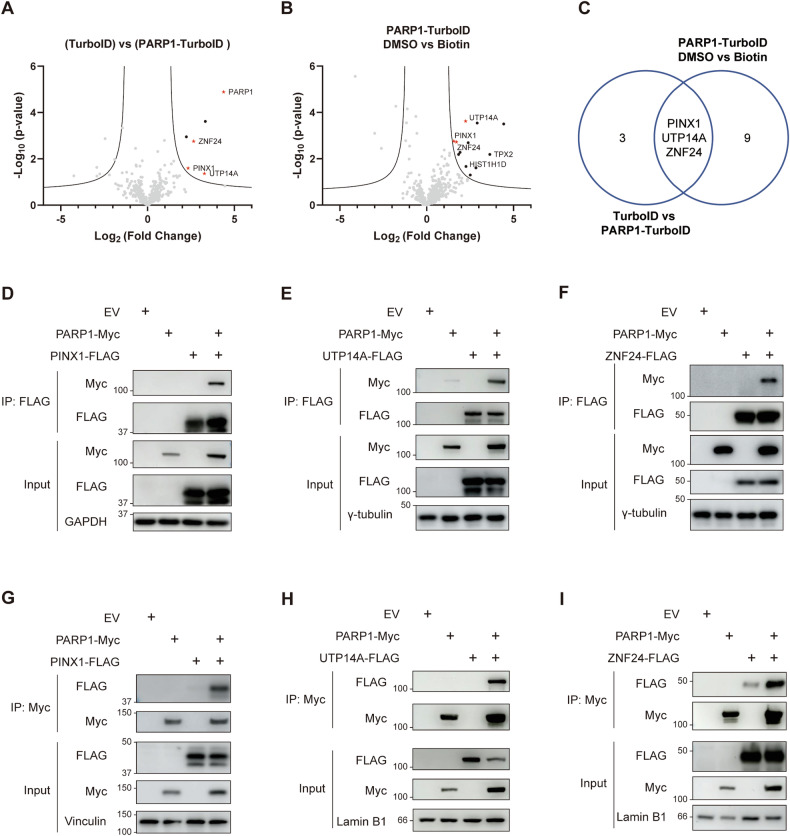
Identification of binding proteins for PARP1 by TurboID. **A** Volcano plot of proximity proteomics of a PARP1-TurboID against a TurboID control. **B** Volcano plot of proximity proteomics of a PARP1-TurboID with or without biotin treatment. The *p*-values were calculated by a two-sample *t*-test. Curves indicate a minimal fold change >2, <−2, and 5% FDR calculated by Perseus. Proteins that show significant proximity to PARP1 are depicted in black dots, while proteins that show significant proximity to PARP1 both in (**A**) and in (**B**) are marked with red stars. **C** Venn diagram of proximity proteins in (**A**) and (**B**). **D**–**F** Exogenous co-IP to confirm the binding between PINX1/UTP14A/ZNF24 and PARP1. PARP1-Myc and PINX1/UTP14A/ZNF24-FLAG constructs were co-transfected into HEK293T cells, and IP of whole cell lysates with anti-FLAG agarose beads was performed. **G**–**I** Reciprocal exogenous co-IP to confirm the binding between PINX1/UTP14A/ZNF24 and PARP1. PARP1-Myc and PINX1/UTP14A/ZNF24-FLAG constructs were co-transfected into HEK293T cells, and IP of whole cell lysates with anti-Myc agarose beads was performed.

To identify novel interaction partners of PARP1, we engineered a fusion of the full-length PARP1 with the TurboID enzyme and two negative controls. The first control omitted the bait protein PARP1, excluding the potential interaction of the TurboID protein itself. However, it bears the drawback of potentially obscuring real interactions due to the high flexibility and activity of free TurboID. The second control omitted exogenous biotin, serving as a complementary measure to address this concern. By conducting the PARP1 TurboID experiment (Details in the Methods section) and analyzing the results with two negative controls, we observed the enrichment of several proteins known to interact with PARP1, such as HIST1H1D and TPX2 [18, 32], particularly in the second comparison (Fig. 1B). Furthermore, we identified three uncharacterized PARP1 binding proteins—UTP14A, PINX1, and ZNF24—enriched in both comparisons (Fig. 1A–C). We conducted biochemical co-immunoprecipitations (co-IPs) to validate these interactions further. As depicted in Fig. 1D–F, Myc-tagged PARP1 co-IPed with all three candidate proteins transiently expressed in HEK293T cells. Reciprocal co-IP experiments provided additional confirmation of these interactions (Fig. 1G–I).

### Loss of PINX1 confers susceptibility to PARP inhibition

**Fig. 2:**
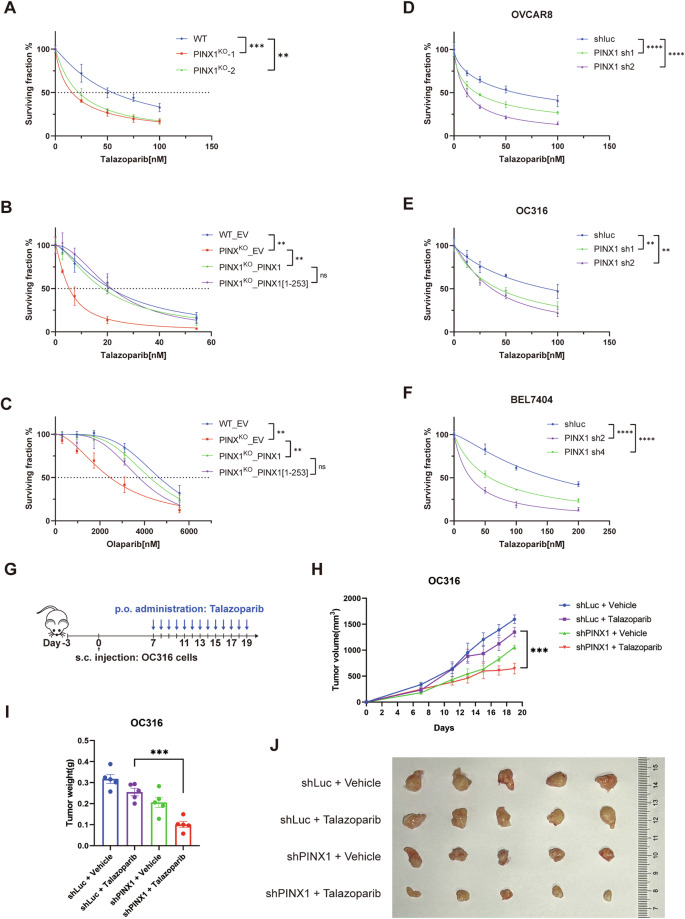
Loss of PINX1 confers susceptibility to PARP inhibition. **A** Sensitivity of WT and PINX1^KO^ Hela cells to Talazoparib. PINX1^KO^-1 and PINX1^KO^-2 represent two distinct PINX1^KO^ single clones derived from WT Hela cells. **B**, **C** Sensitivity of indicated Hela cell lines to Talazoparib (**B**) or Olaparib (**C**). WT_EV, PINX1^KO^_EV, PINX1^KO^_PINX1, and PINX1^KO^_PINX1[1-253aa] are stable cell lines reconstituted with corresponding empty vector (EV), full-length PINX1 or TID domain truncated PINX1 (PINX1[1-253aa]) using lentivirus. **D**–**F** Sensitivity of PINX1 knockdown OVCAR8 (**D**), OC316 (**E**), and BEL7404 (**F**) cells to Talazoparib. shLuc was used as a non-targeted control. **A**–**F** The cell lines were treated with indicated drugs and allowed to grow for 6 days. Cell viability was assessed by the Cell Titer-Glo assay. Surviving fractions were calculated relative to control untreated cells. All dots and error bars represent means ± SDs from at least three independent experiments. Statistical analyses were performed using two-way ANOVA testing. **G** Schematic illustration of the treatment protocol in the OC316 xenograft model. **H** Tumor growth curves of OC316 xenograft tumor-bearing mice for each indicated group (*n* = 5). Significance was determined by an unpaired t-test at the endpoint. Error bars show ±1 SEM. **I** Tumor weight measurement at the endpoint of (**G**). Significance was determined by an unpaired *t*-test. Error bars show ±1 SD. **J** Images of dissected tumor tissues for each indicated group. ns, *p* > 0.05; *, *p* < 0.05; **, *p* < 0.01; ***, *p* < 0.001; ****, *p* < 0.0001.

Considering the practical application value of PARPi in cancer therapy, we investigate whether any of the three newly identified PARP1 interactors could influence the efficacy of PARP inhibitors. Employing shRNAs, we generated knockdown Hela cell lines for each of the three genes. We evaluated their response to the PARP inhibitor Talazoparib through the CellTiter-Glo (CTG) luminescent cell viability assay. Our findings revealed that only the loss of PINX1 rendered the cells hypersensitive to Talazoparib (Supplementary Fig. 1A–F). Notably, this effect was more pronounced in cells where PINX1 was completely knocked out (Fig. 2A and Supplementary Fig. 1G).

PINX1 was first identified as an interacting protein of TRF1 in the telomeric shelterin complex and a potent intrinsic telomerase inhibitor [22, 23]. The TID domain of PINX1 is responsible for its inhibition of telomerase activity [22, 33]. To ascertain whether the impact of PINX1 on the sensitivity to Talazoparib is associated with its role in telomerase inhibition, we introduced both full-length and a C-terminal TID domain-truncated version (PINX1[1–253aa]) form of PINX1 into the PINX1^KO^ cells. Our results demonstrated that re-introducing the full-length or truncated PINX1 eliminated the vulnerability to Talazoparib caused by PINX1 depletion (Fig. 2B and Supplementary Fig. 1H). A similar pattern was also observed with the treatment of another PARP inhibitor, Olaparib (Fig. 2C). These findings suggest that the synergistic effect of PINX1 deletion and PARPi is mediated by an uncharacterized mechanism independent of telomerase inhibition. Furthermore, we observed that this synergistic effect extended beyond Hela cells and could also be observed in OVCAR8, OC316, and BEL7404 cancer cells (Fig. 2D–F and Supplementary Fig. 1I–K), indicating the potential widespread applicability of this mechanism.

To further assess the impact of PINX1 deficiency on the efficacy of PARP inhibitors in vivo, we established a xenograft model in NSG mice using ovarian carcinoma-derived cell line OC316 with or without PINX1 knock-down (Fig. 2G). One week after subcutaneous implantation of OC316 cells, mice were administered by oral gavage daily with either vehicle or Talazoparib. Tumor size was monitored, and the results showed that PINX1 deficient tumors are significantly more sensitive to Talazoparib treatment compared to the PINX1 proficient tumors, suggesting a promising translational possibility (Fig. 2H–J).

### Deletion of PINX1 impairs DNA damage repair

**Fig. 3:**
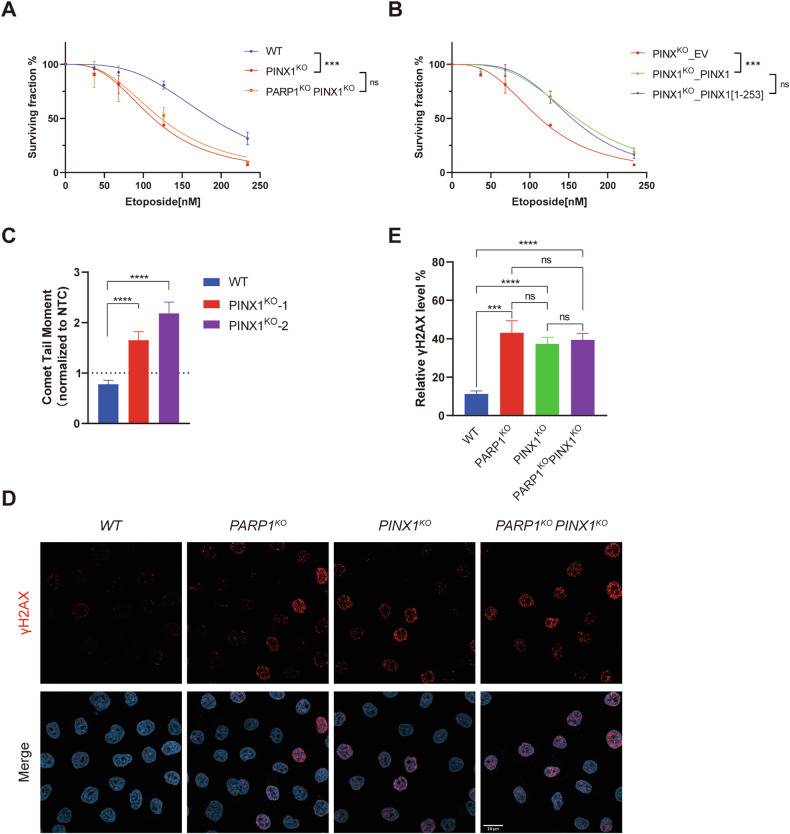
Deletion of PINX1 impairs DNA damage repair. **A** Sensitivity of WT, PINX1^KO^, and PARP1^KO^PINX1^KO^ Hela cells to etoposide. **B** Sensitivity of indicated Hela cell lines to etoposide. PINX1^KO^_EV, PINX1^KO^_PINX1, and PINX1^KO^_PINX1[1–253aa] are stable cell lines reconstituted with corresponding empty vector (EV), full-length PINX1 or TID domain truncated PINX1 (PINX1[1–253aa]) using lentivirus. **A**, **B** The cell lines were treated with indicated drugs and allowed to grow for 6 days. Cell viability was assessed by the Cell Titer-Glo assay. Surviving fractions were calculated relative to control untreated cells. All dots and error bars represent means ± SDs from at least three independent experiments. Statistical analyses were performed using two-way ANOVA testing. **C** Unrepaired DNA strand breaks quantified by alkaline comet assays in the WT and the indicated gene-edited Hela cell lines following treatment with 10 µM etoposide for 30 min and recovery for 30 min. Data plotted are the individual comet tail moments (an arbitrary measure of DNA strand breakage) of ≥116 cells per condition normalized to corresponding no-treatment control (NTC) cells. Significance was determined by an unpaired *t*-test. Error bars show ±1 SEM. **D** Representative images of the levels of γH2AX in WT, PARP1^KO^, PINX1^KO^, and PARP1^KO^PINX1^KO^ Hela cells upon treatment with 10 µM etoposide for 30 min and recovery for 3 h. Red, γH2AX; blue, DAPI. Scale bar, 20 μm. **E** Measurement of remaining γH2AX levels in WT, PARP1^KO^, PINX1^KO,^ and PARP1^KO^PINX1^KO^ Hela cells by immunofluorescence upon treatment with 10 µM etoposide for 30 min and recovery for 3 h. γH2AX intensity was normalized to 0 for untreated cells and 100% for cells without recovery in each cell line. More than 65 cells were analyzed per condition. Significance was determined by an unpaired t-test. Error bars show ±1 SEM. ns, *p* > 0.05; *, *p* < 0.05; **, *p* < 0.01; ***, *p* < 0.001; ****, *p* < 0.0001.

Based on our observation of the synergistic effect of PINX1 deficiency and PARP inhibitors, we aimed to investigate whether this effect resulted from impaired DNA damage repair. To assess the response of PINX1-deficient cells to DNA damage, we initially examined the sensitivity of cells with or without PINX1 to etoposide, a topoisomerase II inhibitor and a DNA damage agent [34], using the CTG assay. As shown in Fig. 3A, PINX1-deficient cells exhibited a notable reduction in cell survival following etoposide treatment compared to wild-type cells, and the double knockout of PINX1 and PARP1 showed no additive effects (Supplementary Fig. 1L). Consistently, stable expression of the full-length and the TID truncated version of PINX1 in the PINX1^KO^ cells showed comparable rescue in cell survival upon the etoposide challenge (Fig. 3B). These results suggest that PINX1-deficient cells are more sensitive to the DNA damage reagent etoposide.

We further quantified unrepaired DNA damage using comet assay and γH2AX immunostaining. The results revealed that WT cells could repair all DNA breaks after treatment with etoposide and recovery, as indicated by an equivalent comet tail moment to the no-treatment control (NTC) cells (Fig. 3C). In contrast, PINX1^KO^ cells retained a significantly higher comet tail moment and γH2AX level (Fig. 3C–E), indicating the presence of more unrepaired DNA damage. Consistent with the CTG assay, the double knockout showed no additive effects in the γH2AX immunostaining assay (Fig. 3D, E), suggesting a collaborative function of PINX1 and PARP1 in DNA damage repair. In conclusion, these findings underscore the essential role of PINX1, along with its binding partner PARP1, in DNA damage repair.

### PINX1 is recruited to DNA damage sites via PARP1 and facilitates the recruitment of XRCC1

**Fig. 4:**
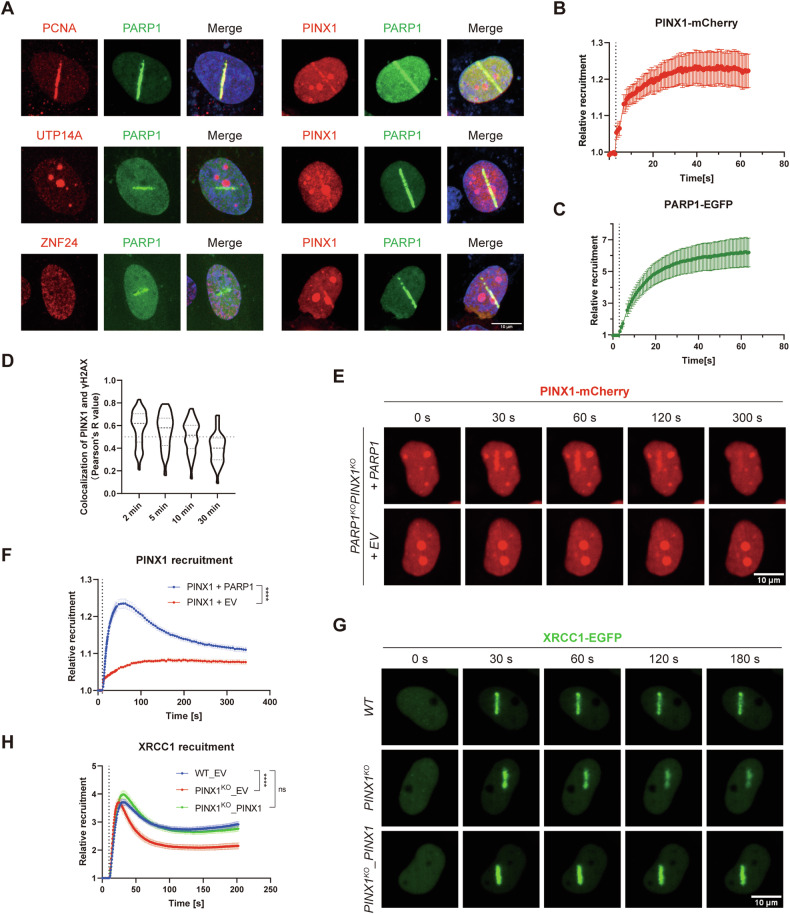
PINX1 is recruited to DNA damage sites via PARP1 and facilitates the downstream recruitment of XRCC1. **A** Representative images of the recruitment of indicated proteins to the laser-induced DNA damage sites. U2OS cells were co-transfected with PARP1–EGFP and PINX1/UTP14A/ZNF24–FLAG constructs, pre-sensitized with Hoechst 33342 before micro-irradiation with a 405 nm laser, and subjected to immunostaining with an anti-FLAG antibody. The enrichment of PARP1–EGFP was used to track the DNA damage sites, and PCNA was used as a positive control for the immunostaining. Red, FLAG; green, EGFP; blue, Hoechst 33342. Scale bar, 10 μm. **B**, **C** Recruitment kinetics of PINX1-mCherry (**B**) and PARP1-EGFP (**C**) to DNA lesions in Hela cells. Hela cells were co-transfected with PINX1-mCherry and PARP1-EGFP constructs and pre-sensitized with Hoechst 33342 before micro-irradiation coupled live-cell imaging. Seventeen nuclei were analyzed, and the intensity of the damaged region was normalized to that of the undamaged region for the relative recruitment. The vertical dotted line indicates the time of laser stimulation. Curves are shown as means ± SEMs. **D** Violin plot of Pearson’s *R* value of colocalization between PINX1–EGFP and γH2AX at indicated time points after laser-induced DNA damage. Hela cells were transfected with PINX1–EGFP and subjected to micro-irradiation as described in Methods and fixed at different time points for immunostaining of γH2AX. More than twenty-nine cells were analyzed per condition. **E** Representative images of the recruitment of PINX1-mCherry to the laser-induced DNA damage sites in PARP1^KO^PINX1^KO^ Hela cells transfected with a PARP1 construct (+PARP1) or corresponding empty vector (+EV). Laser stimulation was performed at 10 s. Scale bar, 10 μm. **F** Recruitment kinetics of PINX1-mCherry to the laser-induced DNA damage sites in PARP1^KO^PINX1^KO^ Hela cells transfected with a PARP1 construct (+PARP1) or corresponding empty vector (+EV). More than sixty nuclei were analyzed per condition. The vertical dotted line indicates the time of laser stimulation. Curves are shown as means ± SEMs. Statistical analyses were performed using two-way ANOVA testing. **G** Representative images of the recruitment of XRCC1–EGFP to the laser-induced DNA damage sites in WT_EV (WT), PINX1^KO^_EV (PINX1^KO^), PINX1^KO^_PINX1 Hela cells. Laser stimulation was performed at 10 s. Scale bar, 10 μm. **H** Recruitment kinetics of XRCC1–EGFP to the laser-induced DNA damage sites in WT_EV (WT), PINX1^KO^_EV (PINX1^KO^), PINX1^KO^_PINX1 Hela cells. More than 31 nuclei were analyzed per condition. The vertical dotted line indicates the time of laser stimulation. Curves are shown as means ± SEMs. Statistical analyses were performed using two-way ANOVA testing. ns, *p* > 0.05; *, *p* < 0.05; **, *p* < 0.01; ***, *p* < 0.001; ****, *p* < 0.0001.

According to the results from comet assay and γH2AX immunostaining, we hypothesized that these newly identified PARP1 interactors, such as PINX1, may be directly involved in the DNA damage repair process along with PARP1. To explore this possibility, we co-transfected FLAG-tagged PCNA, a positive control, or the three candidates with PARP1-EGFP into U2OS cells (Supplementary Fig. 2A, B). Subsequently, these cells were sensitized with Hoechst, subjected to laser micro-irradiation, and underwent subsequent immunostaining. Our results revealed a significant enrichment of PCNA at laser-induced DNA damage sites, co-localizing with enriched PARP1-EGFP, as previously reported [35]. Among the three interactors, we specifically observed a distinguishable enrichment of PINX1 at DNA damage sites, while no such enrichment was detected for UTP14A or ZNF24 (Fig. 4A). We utilized mCherry-fused PINX1 to monitor dynamic recruitment further through laser micro-irradiation coupled with live-cell imaging (Supplementary Fig. 2C, D). The results demonstrated that PINX1 was recruited to DNA lesions as rapidly as PARP1 (Fig. 4B, C), implying possibly direct involvement in DNA damage processing.

Next, we did a time course study to describe how long PINX1 would stay at DNA damage sites, marked by γH2AX or p-ATM. As indicated by a decreased fluorescence enrichment of PINX1 or colocalization Pearson’s *R* value between PINX1 and γH2AX (Fig. 4D and Supplemental Fig. 2C–G), the maximum colocalization of PINX1 at DNA damage sites appeared within 2 minutes after damage, which is consistent with our live-cell imaging data (Fig. 4F and Supplementary Fig. 2I). At the time of 30 min post-damage, the colocalization of PINX1 at the DNA damage sites was disappeared in most of the cells (Pearson’s *R* value < 0.5). These results indicate that PINX1 mainly gets involved in the early stage of DNA damage response.

Considering the association between PARP1 and PINX1, we explored whether the recruitment of these two proteins to DNA damage sites depends on each other. As shown in Fig. 4E, F, the recruitment of ectopically expressed PINX1 was nearly abolished in PARP1 and PINX1 double knockout cells, and the introduction of PARP1 restored the recruitment, indicating a PARP1-dependent recruitment. However, the presence or absence of PINX1 showed no significant impact on the recruitment of PARP1 (Supplementary Fig. 2H).

Subsequently, we delved into the role of PINX1 recruitment in downstream DNA damage response. Given the pivotal partnership between PARP1 and XRCC1 across various DNA repair mechanisms [36], we focused on XRCC1 as our primary candidate, the recruitment of which is largely dependent on PARP1 and is essential for effective downstream repair. Indeed, our experiments revealed that PINX1^KO^ cells exhibited decreased XRCC1 recruitment to DNA damage sites compared to WT, and the re-introduction of PINX1 restored the recruitment (Fig. 4G, H and Supplementary Fig. 2J–K). In summary, these results suggest that PINX1 is recruited to DNA damage sites via PARP1 and facilitates the downstream recruitment of XRCC1.

### PINX1 binds to the ZnF3–BRCT domain of PARP1

**Fig. 5:**
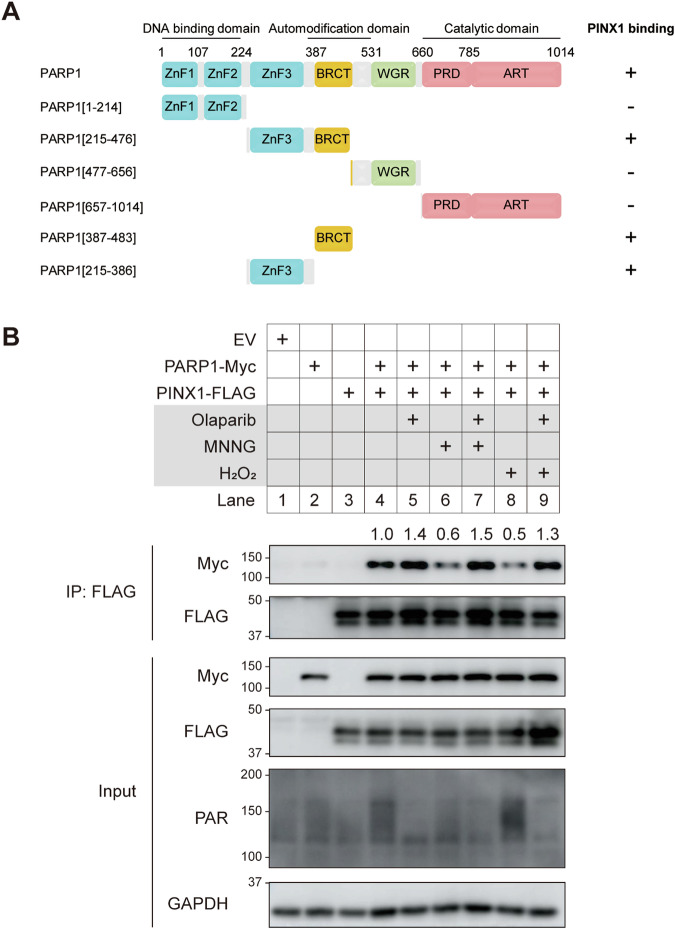
PINX1 binds to the ZnF3-BRCT domain of PARP1. **A** The schematic diagram of PARP1 motifs and mutants used for domain mapping and the summary of their binding ability with PINX1. Numbers indicate the respective amino acid positions. **B** Effect of PARP activation or inhibition on the association between PARP1 and PINX1. PINX1–FLAG was co-transfected with PARP1-Myc into HEK293T cells. Cells were pre-treated with DMSO or 1 µM Olaparib for 40 min before treatment with 50 µM MNNG for 15 min or 2 mM H_2_O_2_ for 5 min. IP of whole cell lysates with anti-FLAG agarose beads was performed.

We conducted a detailed investigation into their interaction to further delineate the binding mechanism between PARP1 and PINX1. Having previously confirmed their interaction in the overexpression system, we first validated that endogenous PARP1 could associate with endogenous PINX1 (Supplementary Fig. 3A). Moving on to map the specific binding region, we designed a series of truncated versions of PARP1 and PINX1, respectively (Fig. 5A; Supplementary Fig. 3C). Our findings revealed that PARP1 primarily binds to PINX1 through its ZNF3 and BRCT domains (Fig. 5A; Supplementary Fig. 3B). Interestingly, almost all tested truncated versions of PINX1 were able to co-IP with PARP1, except for PINX1[1–69aa] (Supplementary Fig. 3D), which is known to be highly unstable in cells and failed to be detected, as reported previously [37]. Therefore, PARP1 associates with the full-length PINX1 via its ZnF3–BRCT domain.

Considering that the BRCT domain serves as a primary auto-modification region when PARP1 is activated by damaged DNA [38, 39], and PAR can either mediate or hinder interactions with other proteins [40, 41], we then explored the binding of PINX1 to PARP1 in the context of DNA damage. When inducing DNA damage with MNNG or H_2_O_2_, we observed a remarkable weakening of the interaction between PARP1 and PINX1. However, treatment in combination with a PARP inhibitor restored the interaction (Fig. 5B, lane 6–9). Notably, treatment with the PARP inhibitor alone enhanced the interaction compared to NTC control (Fig. 5B, lane 4–5). Overall, these results suggest that PINX1 binds to the ZnF3–BRCT domain of PARP1 rather than to the auto-modified PAR, and the highly auto-modified BRCT domain of PARP1 impedes its binding to PINX1.

### PINX1 deficiency results in reduced chromatin-associated PARP1 and expression of genes involved in DNA repair

**Fig. 6:**
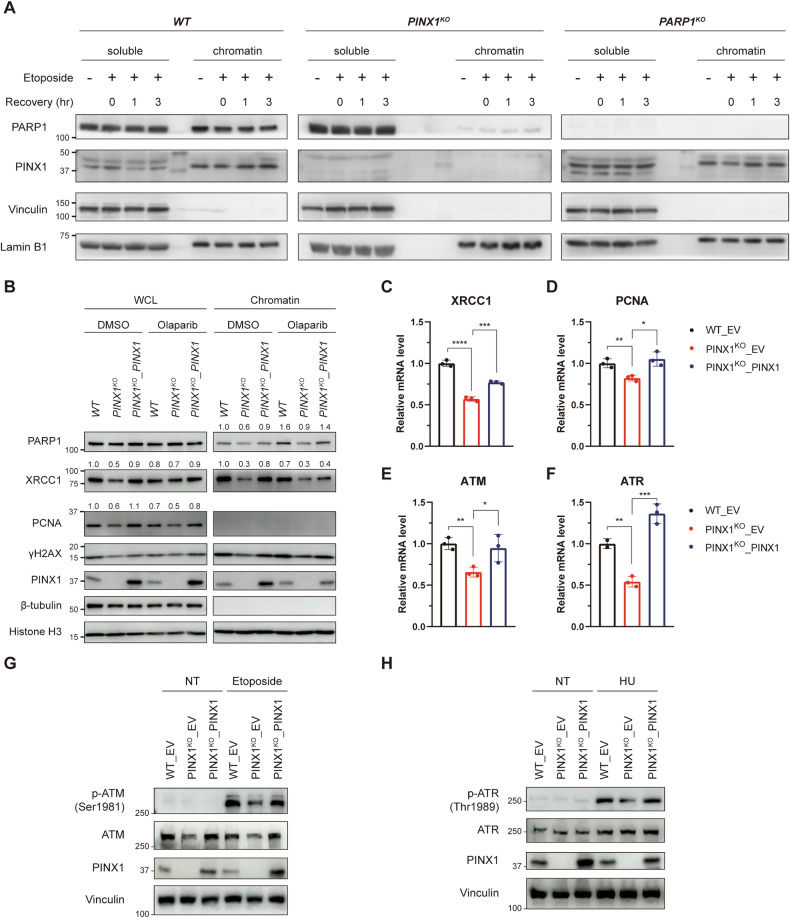
PINX1 deficiency results in reduced chromatin-bound PARP1 and decreased gene expression in DNA repair. **A** Chromatin fractionation of WT, PINX1^KO^, and PARP1^KO^ cells was treated with 10 µM etoposide for 30 min and recovered for the indicated time. Soluble and chromatin fractions were subjected to western blot analysis using the indicated antibodies. **B** Chromatin fractionation of WT_EV (WT), PINX1^KO^_EV (PINX1^KO^), and PINX1^KO^_PINX1 cells treated with DMSO or 10 µM for 1 h. Whole-cell lysates (WCL) and chromatin fractions were subjected to western blot analysis using the indicated antibodies. The data are representative of two independent experiments. **C–F** mRNA levels of XRCC1 (**C**), PCNA (**D**), ATM (**E**), and ATR (**F**) in WT_EV, PINX1^KO^_EV, and PINX1^KO^_PINX1 cells. mRNA expression was measured by qRT-PCR. Error bars show the means ± SDs of samples from at least three replicates, using GAPDH as an endogenous control. **G** WT_EV, PINX1^KO^_EV, and PINX1^KO^_PINX1 cells were treated with 10 μM of etoposide for 17 h and lysed for WB analysis. **H** WT_EV, PINX1^KO^_EV, and PINX1^KO^_PINX1 cells were treated with 4 mM of hydroxyurea (HU) for 23 h and lysed for WB analysis. Significance was determined by an unpaired *t*-test. ns, *p* > 0.05; *, *p* < 0.05; **, *p* < 0.01; ***, *p* < 0.001; ****, *p* < 0.0001.

During our exploration of the effects of PINX1 on cellular DNA repair capacity, we found that PINX1^KO^ cells exhibited a significant decrease in chromatin-associated PARP1 compared to WT cells, both before and after etoposide treatment (Fig. 6A). Given our previous finding that the interaction between PARP1 and PINX1 weakens when PARP1 is activated by DNA damage, we hypothesized that under normal conditions, the constitutive binding of PINX1 and PARP1 might assist in PARP1-chromatin association, exerting corresponding functions that potentially contribute to DNA repair and PARPi resistance. To test this hypothesis further, we performed chromatin fractionations in PINX1-reconstituted KO cells treated with or without PARPi. Interestingly, we observed no significant difference in the total amount of PARP1 protein among WT cells, PINX1^KO^ cells, and PINX1-reconstituted KO cells (Fig. 6B, left, top panel). However, PINX1^KO^ cells exhibited less chromatin-associated PARP1 than WT cells, and upon reconstitution with PINX1, the amount of chromatin-associated PARP1 increased (Fig. 6B, right, top panel). Following PARPi treatment, all cell lines trapped more PARP1 on chromatin, which is consistent with previous reports [9]. Nevertheless, PINX1-deficient cells still displayed less chromatin-associated PARP1 than PINX1-proficient cells (Fig. 6B). This suggests that how PINX1 maintains the chromatin binding of PARP1 is distinct from the trapping effect of PARP inhibitors. We propose that PINX1 preserves the binding of PARP1 to intact chromatin rather than trapping PARP1 on DNA breaks.

Given that PARP1–chromatin association under normal conditions primarily functions in chromatin remodeling and transcription regulation [20, 42, 43], we observed a noticeable reduction in the protein levels of XRCC1, PCNA, and basal γH2AX in both the whole cell lysates and the chromatin-bound fraction of PINX1-deficient cells compared to PINX1-proficient cells (Fig. 6B). We attributed the decrease in basal γH2AX in PINX1-deficient cells to a potential reduction in ATM and ATR, the main upstream kinases responsible for the phosphorylation of the Ser-139 residue of the histone variant H2AX (γH2AX) [44]. We performed an RT-qPCR experiment to address this possibility, revealing reduced mRNA levels of ATM, ATR, XRCC1, and PCNA upon PINX1 loss (Fig. 6C–F). Additional western blot analysis under genotoxic conditions revealed that PINX1 deficient cells exhibit lower levels of ATM or ATR phosphorylation (Fig. 6G, H), indicating impaired DNA damage response. Besides, according to the change in mRNA level, the protein level of ATM is also lower in PINX1 deficient cells, although the level of ATR protein is not significantly affected. Moreover, the reduced PARP1-chromatin association and XRCC1 expression in PINX1-deficient cells were also observed in OC316 cells (Supplementary Fig. 4). Collectively, these results suggest that PINX1 promotes PARP1–chromatin association and the expression of DNA damage repair-related genes, such as XRCC1. This could also account for the DNA damage repair defects and susceptibility to PARP inhibitors in PINX1-deficient cells.

### PINX1 promotes the expression of transcriptional regulators by altering the chromatin binding of PARP1

**Fig. 7:**
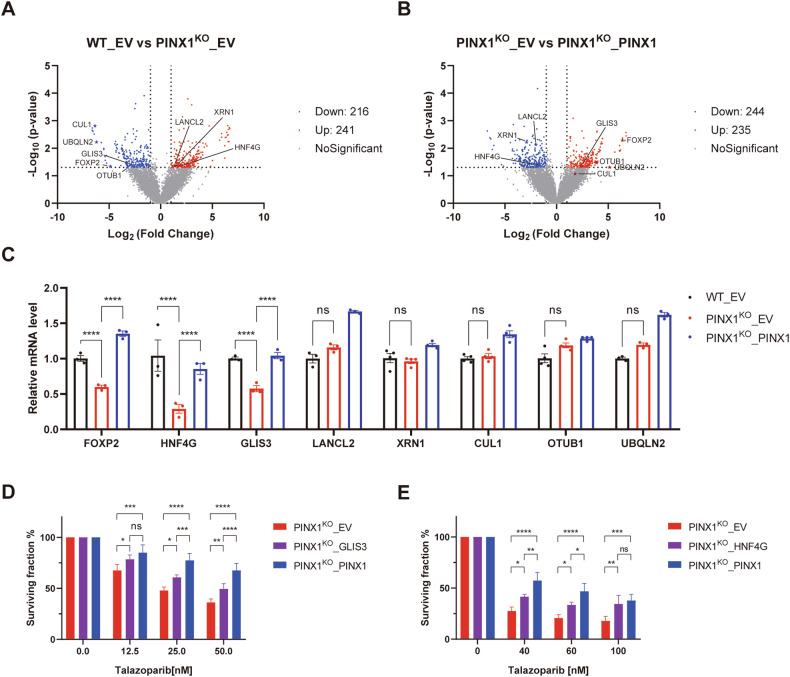
PINX1 promotes the expression of transcriptional regulators by altering the chromatin binding of PARP1. **A** Volcano plot of differential PARP1 binding peaks between PINX1^KO^_EV and WT_EV cells. ChIP-seq was performed using an anti-PARP1 antibody. The differential peaks were determined by more than 2-fold change in read counts and *p*-value ≤ 0.05. Red dots represent a total of 241 upregulated PARP1 binding peaks (Up: 241), blue dots represent a total of 216 downregulated PARP1 binding peaks (Down: 216), gray dots are nonsignificant peaks (No Significant), and differential PARP1 binding peaks adjacent to genes involved in protein expression regulation were labeled and marked by stars. **B** Volcano plot of differential PARP1 binding peaks between PINX1^KO^_PINX1 and PINX1^KO^_EV cells. ChIP-seq was performed using an anti-PARP1 antibody. The differential peaks were determined by more than 2-fold change in read counts and *p*-value ≤ 0.05. Red dots represent a total of 235 upregulated PARP1 binding peaks (Up: 235), blue dots represent a total of 244 downregulated PARP1 binding peaks (Down: 244), gray dots are nonsignificant peaks (No Significant), and differential PARP1 binding peaks adjacent to genes involved in protein expression regulation were labeled and marked by stars. **C** mRNA levels of genes with differential PARP1 binding upon PINX1 depletion in WT_EV, PINX1^KO^_EV, PINX1^KO^_PINX1 cells. mRNA expression was measured by qRT-PCR. Error bars show the means ± SDs of samples from at least three replicates, using GAPDH as an endogenous control. Significance was determined by an unpaired *t*-test. **D** GLIS3 supplementation in PINX1^KO^ cells partially rescued the susceptibility to Talazoparib. PINX1^KO^_EV, PINX1^KO^_GLIS3, and PINX1^KO^_PINX1 are stable cell lines reconstituted with corresponding empty vector (EV), GLIS3, or PINX1 using lentivirus. **E** HNF4G supplementation in PINX1^KO^ cells partially rescued the susceptibility to Talazoparib. PINX1^KO^_EV, PINX1^KO^_HNF4G, and PINX1^KO^_PINX1 are stable cell lines reconstituted with corresponding empty vector (EV), HNF4G, or PINX1 using lentivirus. Error bars show the means ± SDs of samples from at least three replicates. Significance was determined by an unpaired *t*-test. ns, *p* > 0.05; *, *p* < 0.05; **, *p* < 0.01; ***, *p* < 0.001; ****, *p* < 0.0001.

As outlined above, we observed a reduced (a) chromatin-bound PARP1 protein, and (b) expression of some genes involved in DNA repair at mRNA level in PINX1 deficient cells. Given the potential impact of chromatin-bound PARP1 in transcriptional regulation, we wondered whether above mentioned PINX1-related expression change is mediated by the PINX1-PARP1 axis. To investigate this possibility, we conducted the chromatin immunoprecipitation (ChIP) sequencing analysis to identify the specific alterations in the presence of PINX1 on the binding of PARP1 to chromatin. We enriched PARP1-bound chromatin using lysates from WT, PINX1^KO^, or PINX1-reconstituted KO cells with an anti-PARP1 antibody and subjected them to sequencing. The results revealed changes in PARP1 binding at numerous chromatin sites in cells with or without PINX1 (Fig. 7A, B). We identified several sites where PARP1 binding increased or decreased after PINX1 depletion, and these changes were restored upon reintroducing PINX1. Thus, PINX1 was found to regulate the binding of PARP1 to these sites. By mapping these loci onto the genome, we obtained a list of adjacent protein-coding genes and speculated that the expression of these genes might be regulated by PINX1–PARP1 binding. Although above mentioned PINX1-associated DNA damage repair-related genes, such as XRCC1 were not included in the list, we found numerous genes involved in gene expression or protein degradation, as marked in Fig. 7A, B. We suspected that the possible expression change in these genes might account for at least part of the phenotypes in PINX1-deficient cells.

We conducted a qPCR experiment to verify whether the transcription levels of these genes changed in cells with or without PINX1. The results demonstrated that the mRNA levels of three transcriptional regulators, FOXP2, HNF4G, and GLIS3, significantly decreased without PINX1 but increased when PINX1 was restored (Fig. 7C). Furthermore, supplementation with GLIS3 or HNF4G in PINX1-deficient cells partially rescued the susceptibility to PARP inhibitors resulting from PINX1 deletion (Fig. 7D, E and Supplementary Fig. 5A, B). In contrast, no significant difference was observed in FOXP2 supplementation (Supplementary Fig. 5C, D). In conclusion, we propose that PINX1 regulates the PARP1–chromatin association and promotes the expression of certain transcriptional regulators, such as GLIS3 and HNF4G, ultimately enhancing cell resistance to PARP inhibitors.

## Discussion

PARP inhibitors have demonstrated effectiveness in treating HR-deficient cancers and show potential for broader application due to the wide range of functions carried out by PARP1 in cells. Approving PARP inhibitors for cancer treatment in specific contexts, irrespective of BRCA status [19], has prompted further exploration of their therapeutic potential and underlying mechanisms. Notably, several emerging synthetic lethal partners are interactors and direct regulators of PARP1 [12, 16–18, 45, 46]. Seeking to expand our understanding of the biological roles of PARP1 and the potential of PARP inhibitors, we identified PINX1, UTP14A, and ZNF24 as novel PARP1 interactors using TurboID combined with co-IP validation (Fig. 1). The absence of PINX1 rendered Hela cells susceptible to PARP inhibitors, a phenomenon not only confirmed in OVCAR8, OC316, and BEL7404 cancer cell lines but also validated in vivo in xenograft mouse models (Fig. 2), suggesting PINX1 as a potential target for cancer therapy. Interestingly, TID domain-truncated PINX1, known to lose its telomerase inhibitory activity [33], retained its ability to associate with PARP1 and ultimately rescued vulnerability to PARP inhibitors caused by PINX1 deficiency (Fig. 2B, C). This indicates that PINX1 is involved in this process as a PARP1 interacting protein rather than a telomerase inhibitor.

As expected, PINX1-deleted cells exhibited impaired DNA damage repair capacity (Fig. 3). Both TID-truncated and full-length PINX1 rescued this phenotype. To understand the specific mechanism, we investigated whether PINX1 directly participates in DNA damage response. Laser micro-irradiation experiments revealed rapid recruitment of PINX1 to DNA damage sites by PARP1 and a gradual dissociation in 30 min, while UTP14A and ZNF24, also interacting with PARP1, were not enriched (Fig. 4A–F). At DNA damage sites, PINX1 assisted in recruiting the downstream repair factor XRCC1 (Fig. 4G, H), which could explain the impaired DNA damage response in PINX1 deficient cells.

Furthermore, we found that PARP1 binds to PINX1 through its ZnF3–BRCT domain (Fig. 5A), which is usually modulated by auto-modification. Given the quite fast, massive, and dynamic nature of PARylation, this post-translational modification is known to regulate different interactions [47]. For example, PARP1 could be recruited to and activated by damaged DNA, whereas the excessive auto-modification of PARP1 facilitates its dissociation from DNA lesions [1, 4]. In addition, the accessibility of the Argonaute/miRNA complex to its corresponding mRNA is hindered by an increase in local PARylation on multiple proteins that bind to the target, resulting in the offset of miRNA silencing [48]. In the context of our findings, we observed an immediate and dynamic recruitment of PINX1 to laser-induced DNA lesions in the early stage of DNA damage response (Fig. 4A–F). However, when PARP1 is hyperactivated by MNNG or H_2_O_2_ treatment, excessive PARylation hinders the interaction between PARP1 and PINX1. Co-treatment with PARP inhibitor olaparib, which inhibits the auto-PARylation of PARP1, restored this interaction (Fig. 5B). These results highly suggest that the interaction between PARP1 and PINX1 is dynamically modulated by the level of PARylation.

PARP1 primarily binds to DNA lesions through its Zinc finger domains [4, 49]. Additionally, PARP1 constitutively binds to chromatin in its inactive form without DNA damage [50], a process related to gene expression regulation and other functions [20]. We observed that PINX1 deficiency significantly reduced chromatin-bound PARP1 and decreased transcription and protein levels of repair-related genes such as XRCC1 and ATM within cells (Fig. 6). This could explain the impaired DNA damage repair capacity of PINX1-deficient cells. Through ChIP-seq, we found that the absence of PINX1 caused various changes in the binding of PARP1 to numerous chromatin sites (Fig. 7A, B). However, regulatory elements for PINX1-associated DNA repair-related genes, such as XRCC1, were not included. It indicates that the level of those PINX1-associated DNA repair genes is not directly transcriptionally regulated by PINX1–PARP1. However, within the protein-coding genes with differential PARP1 binding in the presence/absence of PINX1, and by focusing on genes involved in protein expression regulation, we finally found that the transcription levels of GLIS3, HNF4G, and FOXP2, three transcription regulatory factors, decreased in cells lacking PINX1 (Fig. 7C). Supplementing GLIS3 or HNF4G in PINX1-deficient cells can partially rescue the susceptibility to PARP inhibitors caused by PINX1 deficiency (Fig. 7D, E and Supplementary Fig. 5A, B). At the same time, no significant changes were observed by supplementing FOXP2 (Supplementary Fig. 5C, D). This indicates that the reduction of GLIS3 and HNF4G caused by PINX1 deficiency partially accounts for the response of cells to PARP inhibitors, while other reasons include that PINX1 directly participates in DNA damage response (Fig. 4). Besides, since most peaks with differential PARP1 binding are located in distal intergenic regions (Supplementary Table S2), the influence on non-coding DNA can not be excluded.

The ZnF3 domain of PARP1 is reported to be crucial for mediating inter-domain contact, chromatin compression activity, and transcriptional inhibition of PARP1 [51]. Meanwhile, the BRCT domain of PARP1 has recently been identified as a novel domain that binds to intact DNA through a ‘monkey bar’ mechanism facilitating DNA transfer of PARP1 [52]. PINX1 may assist in chromatin binding and transcriptional regulation of PARP1 by binding to these two domains. Moreover, PINX1 has been reported to regulate transcription through transcription factors such as AR and ERα [26, 53]. Thus, we do not exclude the possibility that PINX1 holds cellular stress capacity independently of PARP1. Differences in the composition of these mechanisms in different cancer cells may also explain the reported paradoxical function of PINX1 in either promoting or inhibiting cancer progression, requiring further research to clarify.

**Fig. 8:**
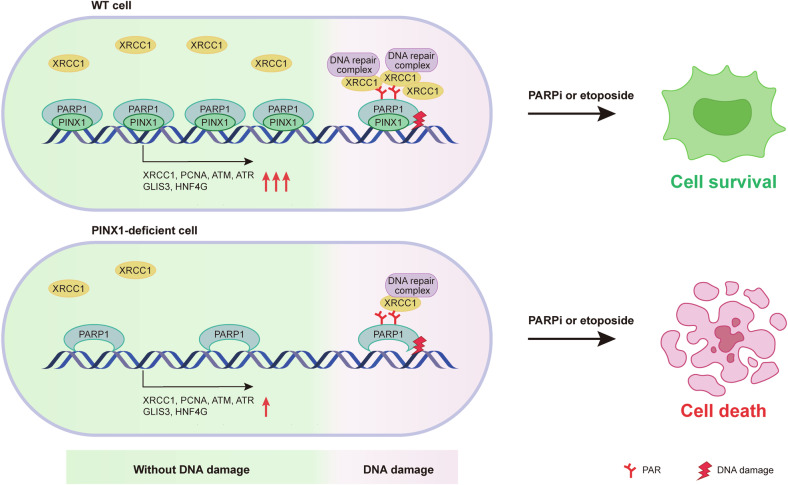
Model for PINX1 promoting cellular response to DNA damage and PARP inhibitors. PINX1 acts as a multifaceted partner of PARP1 that promotes cellular response to DNA damage and PARP inhibitors. Under normal conditions, PINX1 constitutively binds to PARP1, aiding in chromatin association and transcriptional regulation of PARP1, enhancing the expression of DNA repair genes such as XRCC1 and transcriptional regulatory factors such as GLIS3, ensuring cellular DNA damage repair capacity. At the time of DNA damage, PINX1 is rapidly recruited to the damage sites via PARP1, facilitating the recruitment of early repair factor XRCC1 and downstream DNA repair processes. Both mechanisms contribute to cellular defense against PARP inhibitors.

In summary, our data support a model in which PINX1 acts as a multifaceted partner of PARP1, promoting cellular response to DNA damage and PARP inhibitors in multiple ways (Fig. 8). During DNA damage, PINX1 is rapidly recruited to the damaged sites via PARP1, facilitates the recruitment of early repair factor XRCC1, and gradually dissociates from the damaged sites due to the auto-modification of PARP1 at the binding site. Under normal conditions, PINX1 constitutively binds to PARP1, aiding in chromatin association and transcriptional regulation of PARP1, enhancing the expression of DNA repair genes such as XRCC1 and transcriptional regulatory factors such as GLIS3. Furthermore, we demonstrated that PINX1 deficiency leads to susceptibility of cancer cells to PARP inhibitors both in vitro and in vivo, suggesting that its inhibition combined with PARP inhibitors can serve as a potential therapeutic strategy.

### Supplementary information

Supplementary Figures and Legends

Supplementary Table S1

Supplementary Table S2

uncropped western blots

## Data Availability

The mass spectrometry proteomics data have been deposited to the ProteomeXchange Consortium (http://proteomecentral.proteomexchange.org) via the iProX partner repository with the dataset identifier PXD049100. The raw and processed ChIP-seq data have been deposited into the NCBI Sequence Read Archive under the accession number PRJNA1078510. Any additional information required to reanalyze the data reported in this work is available from the Lead Contact upon request.
