# Supplementary material for: PINX1 loss confers susceptibility to PARP inhibition in pan-cancer cells
Source: Cell Death Dis. 2024 Aug 22;15(8):610. doi: 10.1038/s41419-024-07009-6 (PMC11341912; doi:10.1038/s41419-024-07009-6)
Supplement: Supplementary file 1 — Supplementary Figures and Legends [file 41419_2024_7009_MOESM1_ESM.docx]

## Supplementary Figures


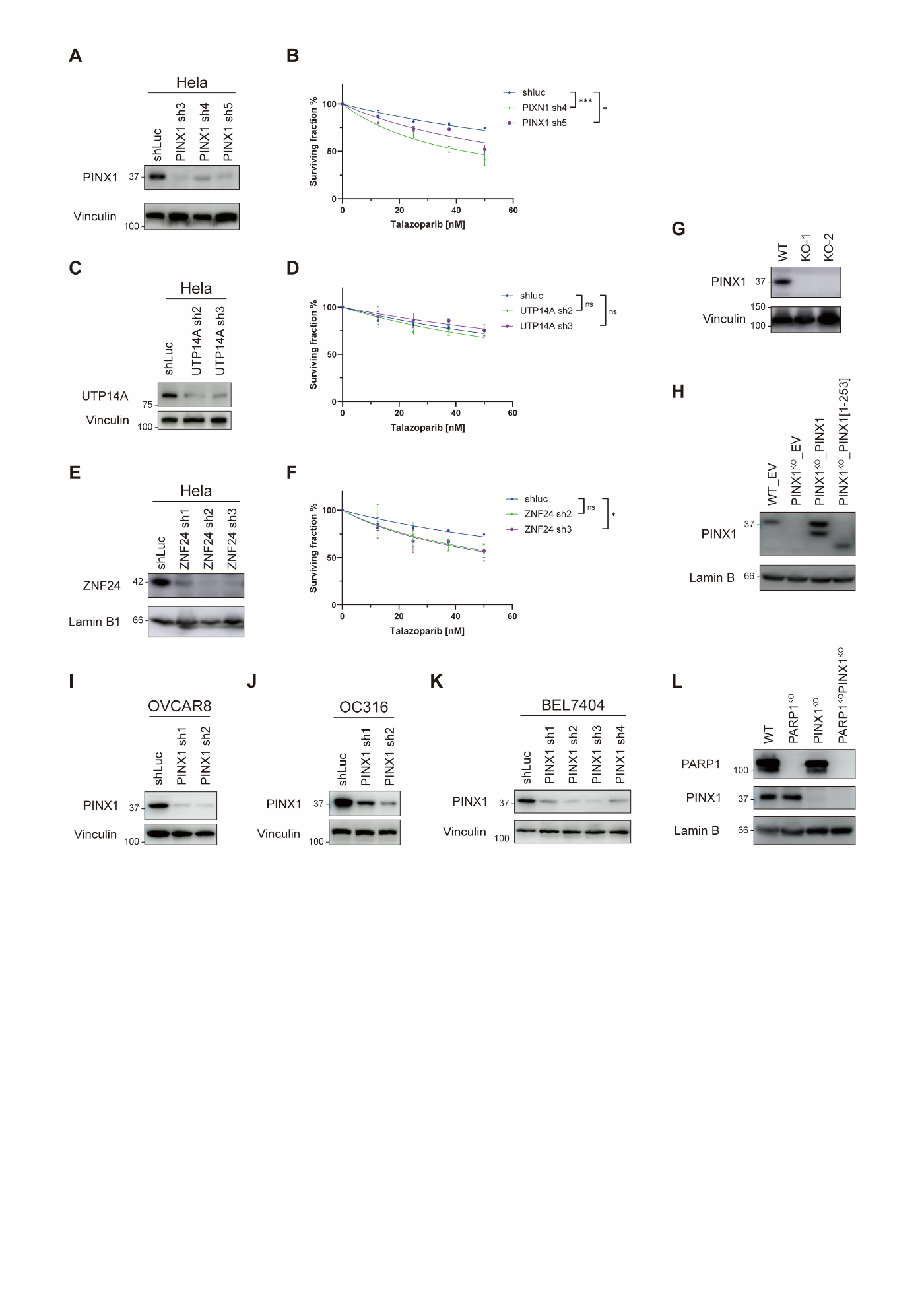


### Supplementary Fig. 1

(A) WB validation of PINX1 knockdown in Hela cells. shLuc was used as a non-targeted control.

(B) Sensitivity of PINX1 knockdown Hela cells to talazoparib.

(C) WB validation of UTP14A knockdown in Hela.

(D) Sensitivity of UTP14A knockdown Hela cells to talazoparib.

(E) WB validation of ZNF24 knockdown in Hela.

(F) Sensitivity of ZNF24 knockdown Hela cells to talazoparib.

(G) WB validation of 2 independent Hela PINX1 knockout single clone.

(H) WB validation of PINX1 knockout Hela cells rescued with empty vector (EV), full-length PINX1 or TID domain truncated PINX1 (PINX1[1-253]).

(I-K) WB validation of PINX1 knockdown in OVCAR8 (I), OC316 (J), BEL7404 (K) cells. (L) WB validation of PARP1^KO^, PINX1^KO^, or PARP1^KO^PINX1^KO^ double knockout Hela cells.

Statistical analyses were performed using two-way ANOVA testing. ns, p > 0.05; *, p < 0.05; **, p < 0.01; ***, p < 0.001; ****, p < 0.0001.


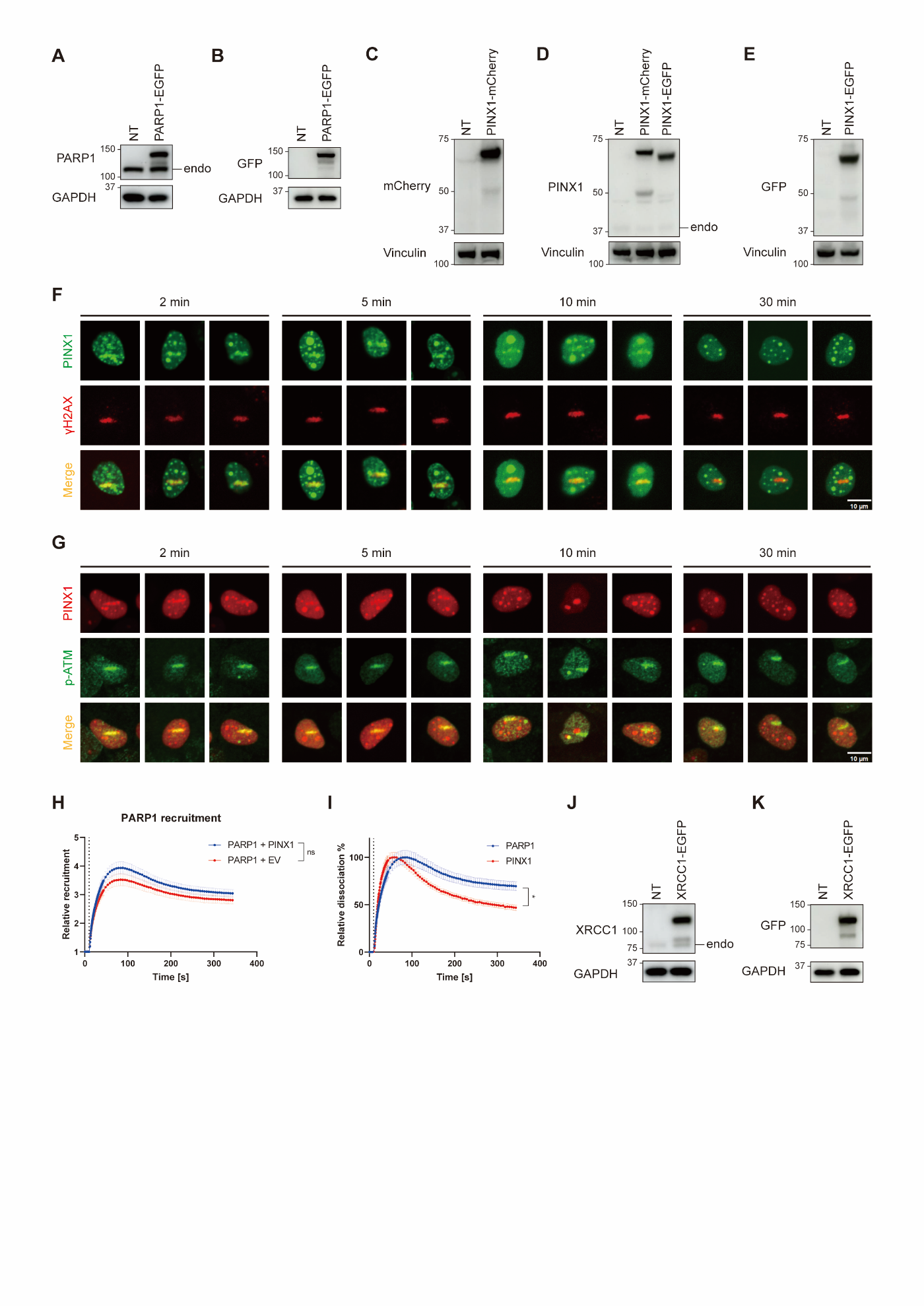


### Supplementary Fig. 2

(A-B) WB validation of the correct expression of PARP1-EGFP fusion protein with the PARP1 antibody (A) and the GFP antibody (B). endo indicates the endogenous expression of PARP1.

(C-E) WB validation of the correct expression of PINX1-mCherry or PINX1-EGFP fusion protein with the mCherry antibody (C), the PINX1 antibody (D), or the GFP antibody (E). endo indicates the endogenous expression of PINX1.

(F) Representative images of Figure 4D, showing the localization of PINX1-EGFP at DNA damage sites marked by γH2AX at indicated time after laser-induced DNA damage. Hela cells were transfected with PINX1-EGFP and subjected to micro-irradiation as described in methods, and fixed at different time points for immunostaining of γH2AX. Green, PINX1-EGFP; red, γH2AX. Scale bar, 10 μm.

(G) Representative images of the localization of PINX1-mCherry at DNA damage sites marked by p-ATM at indicated time after laser-induced DNA damage. Hela cells were transfected with PINX1-mCherry and subjected to micro-irradiation as described in methods, and fixed at different time points for immunostaining of p-ATM. Red, PINX1-mCherry; green, p-ATM. Scale bar, 10 μm.

(H) Recruitment kinetics of PARP1-EGFP to the laser-induced DNA damage sites in PARP1^KO^PINX1^KO^ Hela cells transfected with a PINX1 construct (+ PINX1) or corresponding empty vector (+ EV). ≥ 48 nuclei were analyzed per condition. The vertical dotted line indicates the time of laser stimulation. Curves are shown as means ± SEMs. Statistical analyses were performed using two-way ANOVA testing. ns, p > 0.05; *, p < 0.05; **, p < 0.01; ***, p < 0.001; ****, p < 0.0001.

(I) Dissociation kinetics of PARP1-EGFP and PINX1-mCherry from the laser-induced DNA damage sites in Hela cells. To describe the dissociation kinetics, the max relative recruitment was normalized to 100%, and the initial value was defined as 0. ≥ 48 nuclei were analyzed per condition. The vertical dotted line indicates the time of laser stimulation. Curves are shown as means ± SEMs. Statistical analyses were performed using two-way ANOVA testing. ns, p > 0.05; *, p < 0.05; **, p < 0.01; ***, p < 0.001; ****, p < 0.0001.

(J-K) WB validation of the correct expression of XRCC1-EGFP fusion protein with the XRCC1 antibody (J) and the GFP antibody (K). endo indicates the endogenous expression of XRCC1.


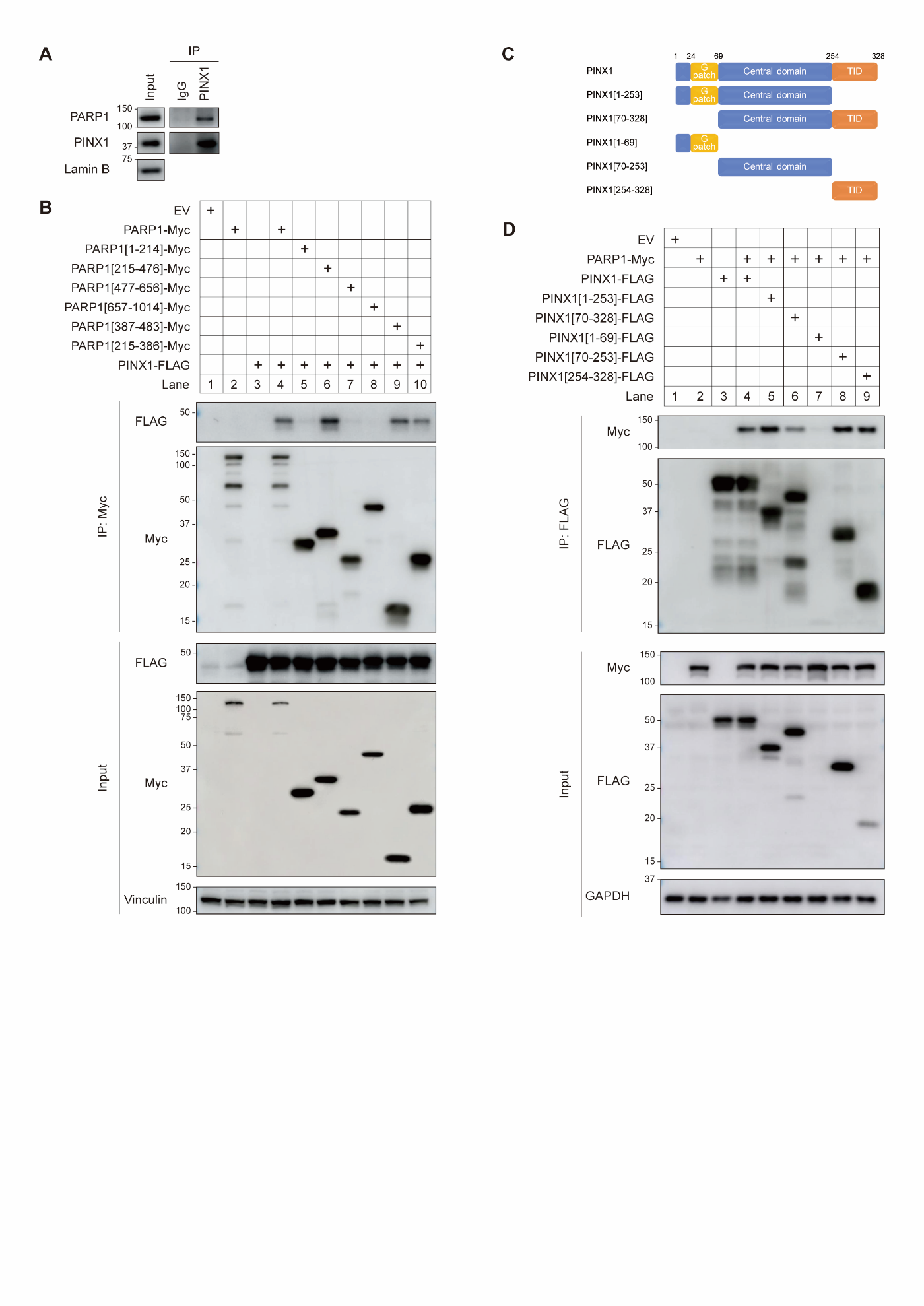


### Supplementary Fig. 3

(A) Western blot validation of the association between PARP1 and PINX1 using an antibody against endogenous PINX1 or an IgG control for immunoprecipitation.

(B) Domain mapping for the region on PARP1 responsible for PINX1-binding. PINX1-FLAG was cotransfected with PARP1-Myc or its deletion mutants into HEK293T cells. IP of whole cell lysates with anti-Myc agarose beads was performed.

(C) Schematics of full-length PINX1 and mutants used for domain mapping. Numbers indicate the respective amino acid positions.

(D) Domain mapping for the region on PINX1 responsible for PARP1-binding. PARP1-Myc was cotransfected with PINX1-FLAG or its deletion mutants into HEK293T cells. IP of whole cell lysates with anti-FLAG agarose beads was performed.


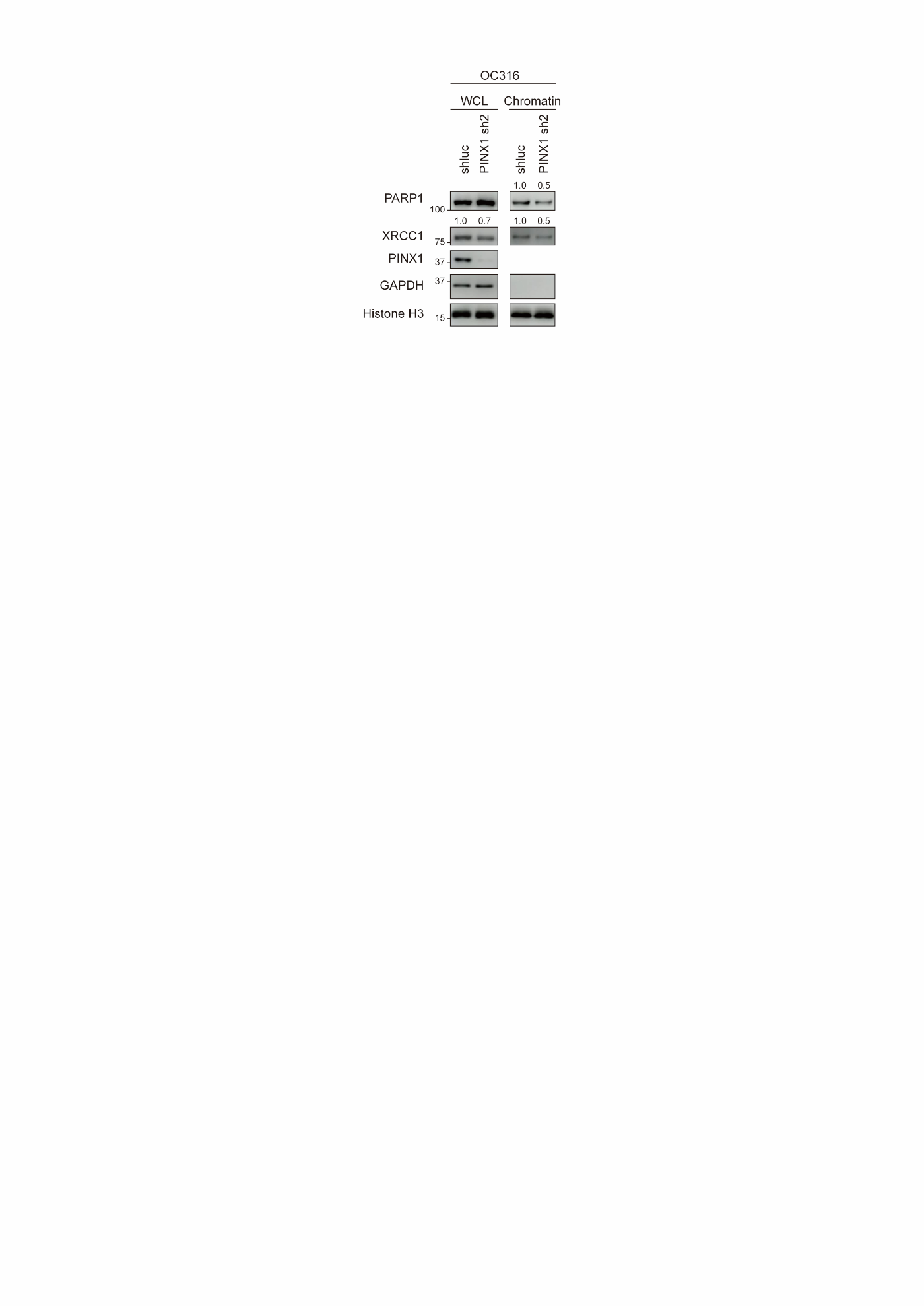


### Supplementary Fig. 4

Chromatin fractionation of PINX1 knockdown (PINX1 sh2) or control (shLuc) OC316 cells. Whole cell lysates (WCL) and chromatin fractions were subjected to western blot analysis using the indicated antibodies. The data is representative of two independent experiments.


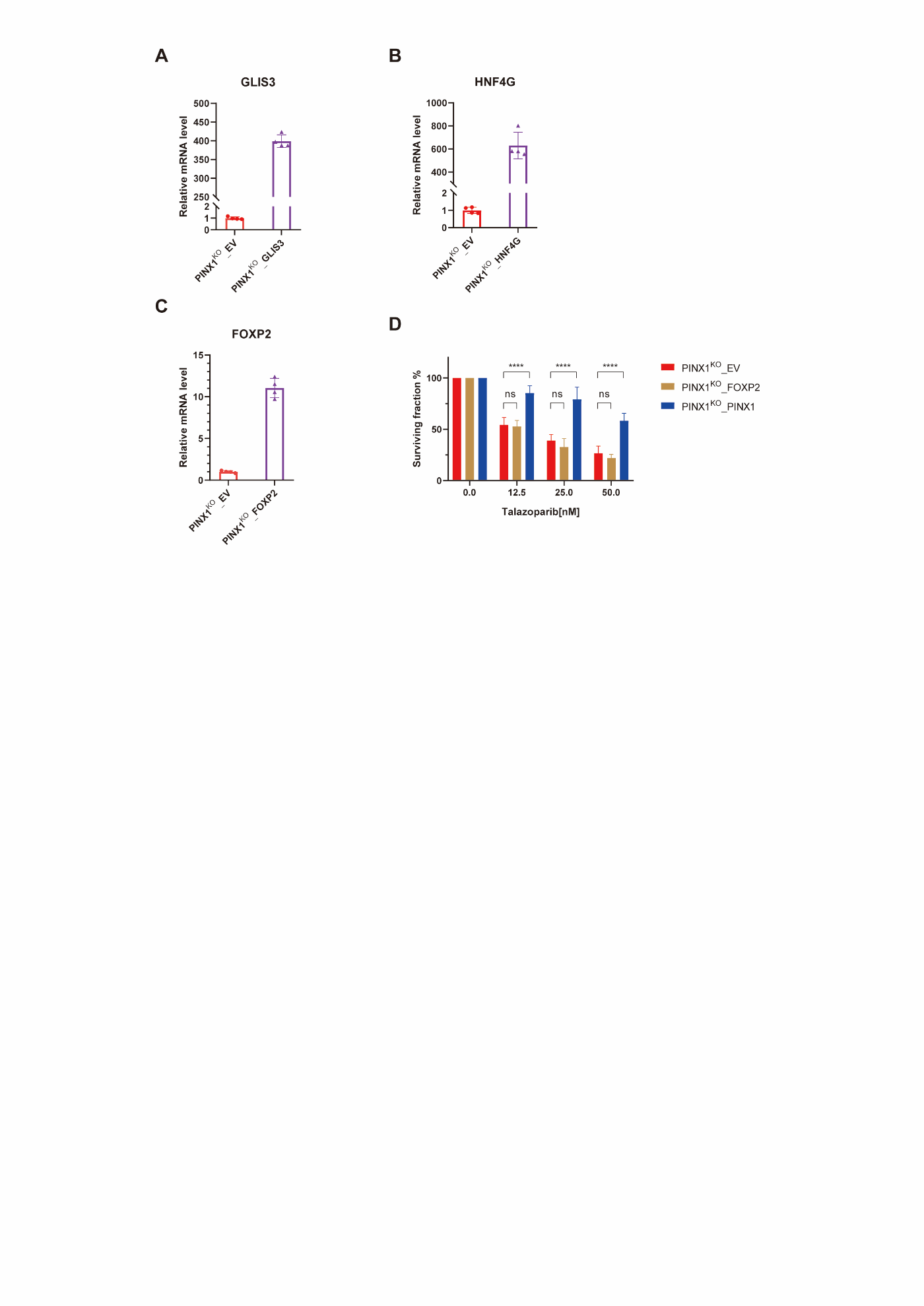


### Supplementary Fig. 5

(A-C) qPCR validation of PINX1^KO^ cells rescued with GLIS3 (A), HNF4G (B) or FOXP2 (C).

(D) FOXP2 supplementation in PINX1^KO^ cells failed to rescue the susceptibility to talazoparib. PINX1^KO^_EV, PINX1^KO^_FOXP2, and PINX1^KO^_PINX1 are stable cell lines reconstituted with corresponding empty vector (EV), FOXP2, or PINX1 using lentivirus. Error bars show the means ± SDs of samples from at least three replicates. Significance was determined by an unpaired t-test. ns, p > 0.05; *, p < 0.05; **, p < 0.01; ***, p < 0.001; ****, p < 0.0001.
