# Supplementary material for: PINX1 loss confers susceptibility to PARP inhibition in pan-cancer cells
Source: Cell Death Dis. 2024 Aug 22;15(8):610. doi: 10.1038/s41419-024-07009-6 (PMC11341912; doi:10.1038/s41419-024-07009-6)

Figure 1D

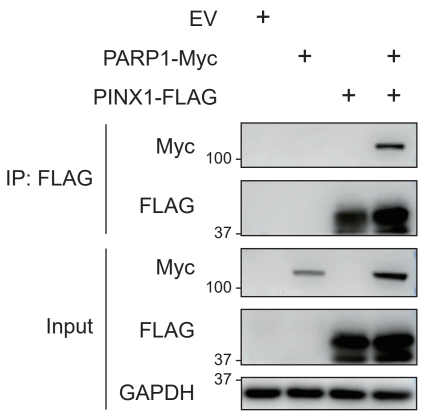

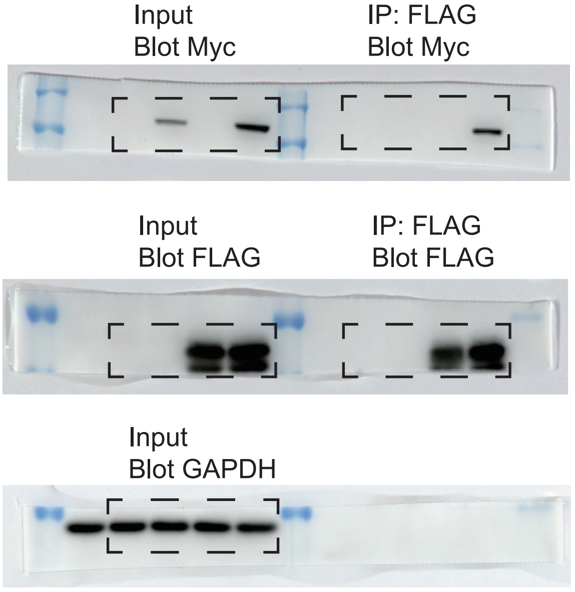

Figure 1E

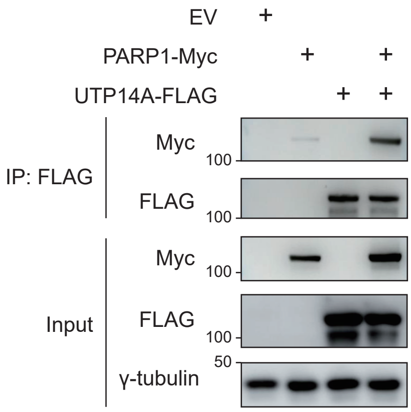

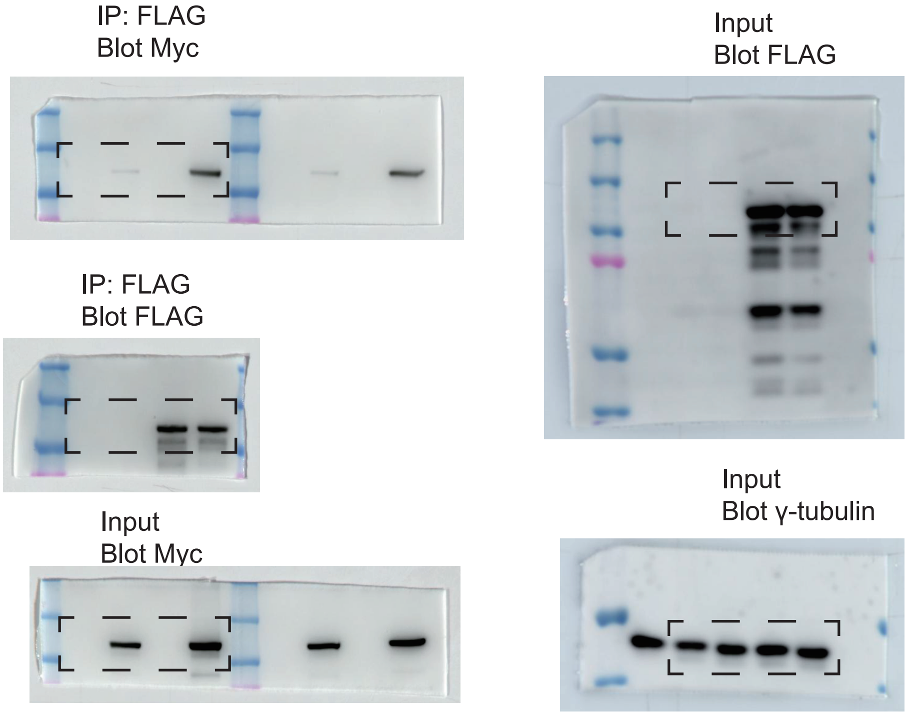

Figure 1F

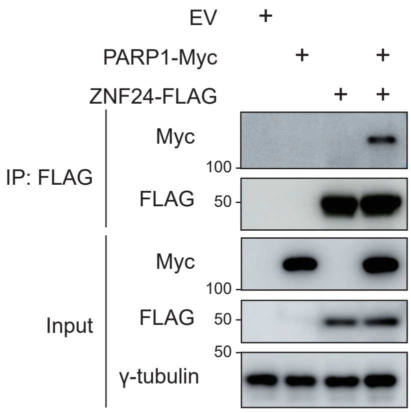

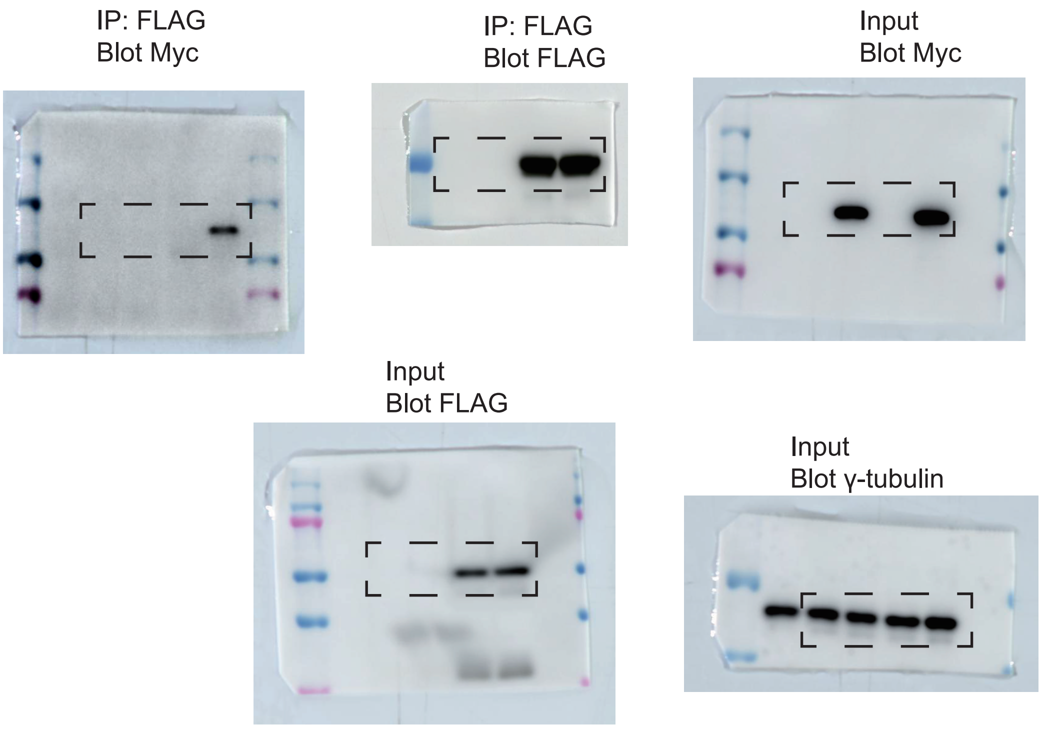

Figure 1G

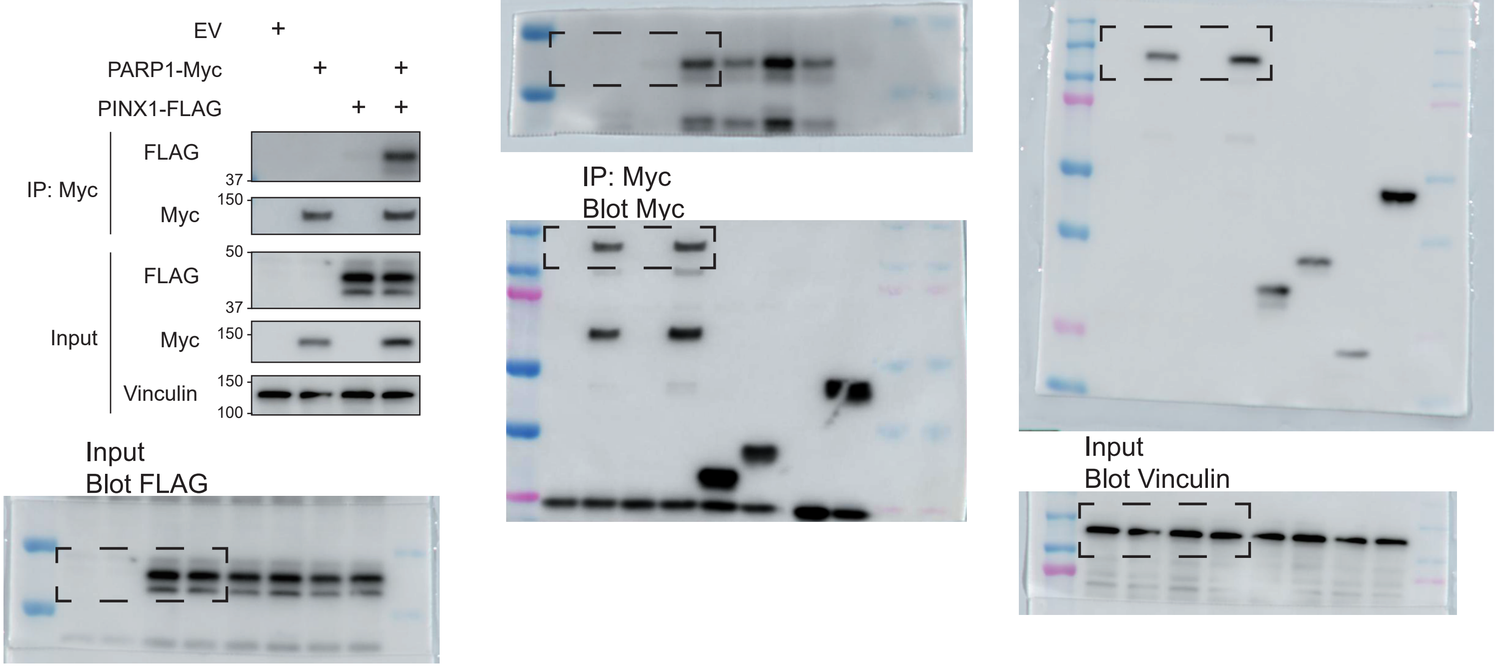

Figure 1H

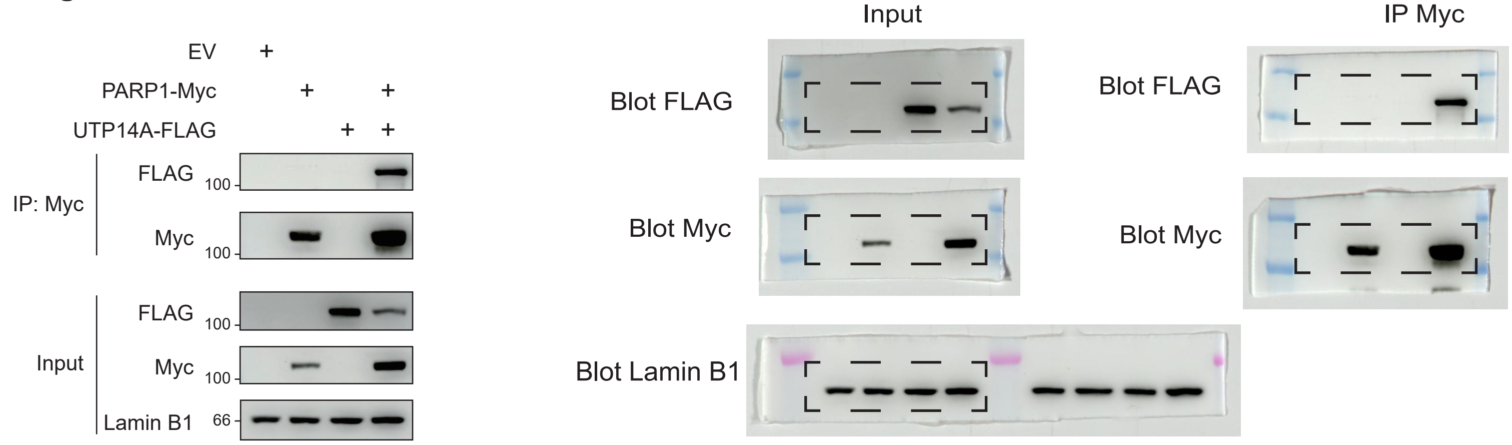

Figure 1I

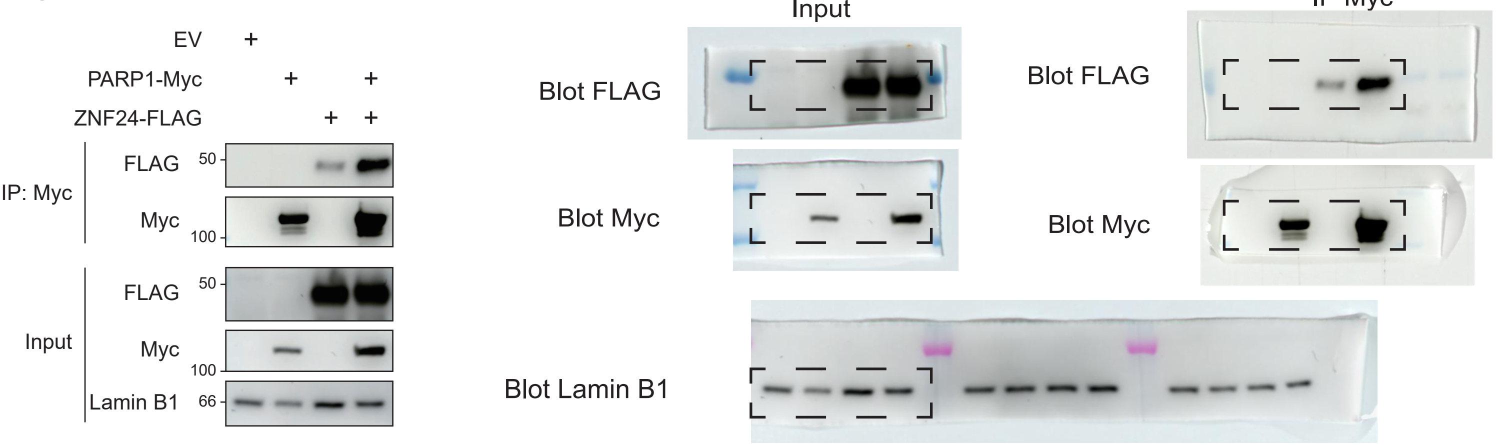

Supplementary Fig. 1A

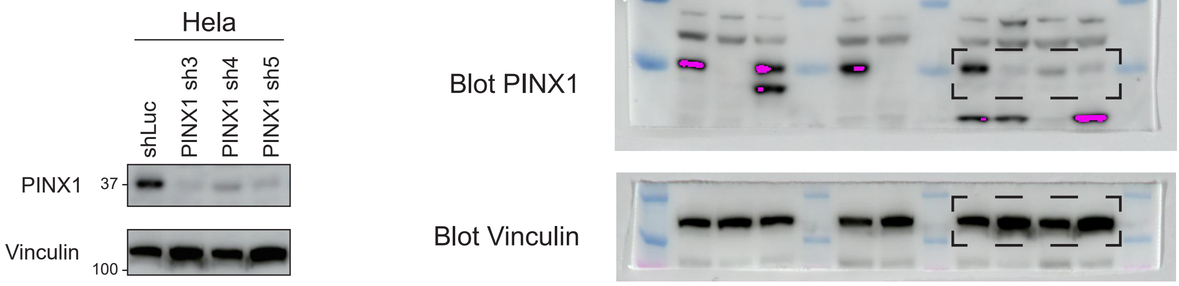

Supplementary Fig. 1C

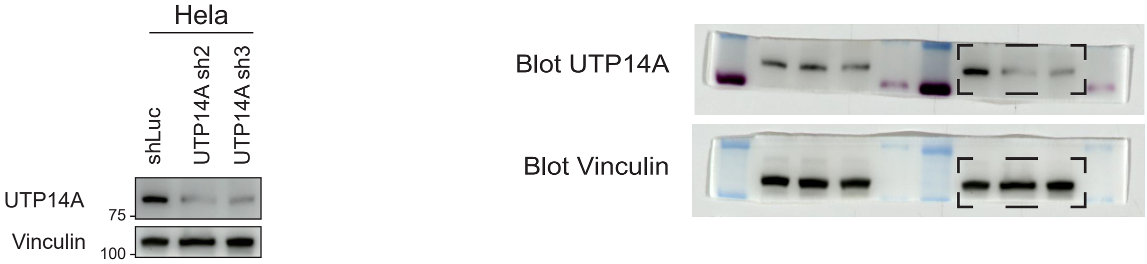

Supplementary Fig. 1E

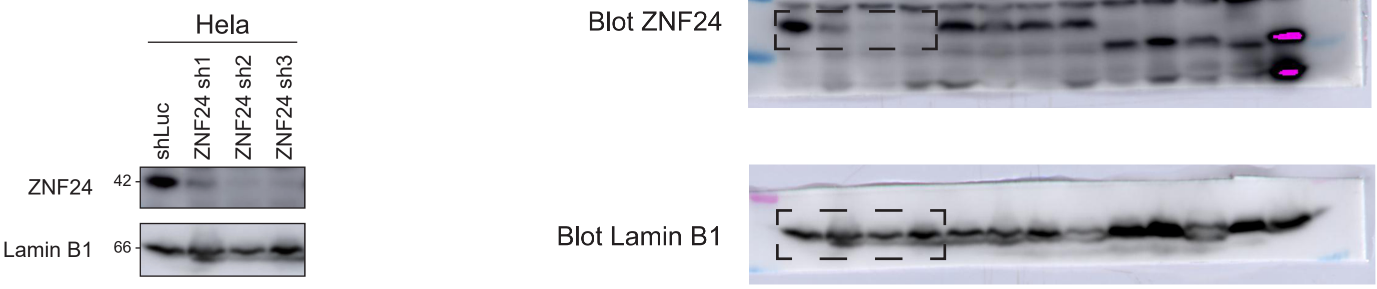

Supplementary Fig. 1G

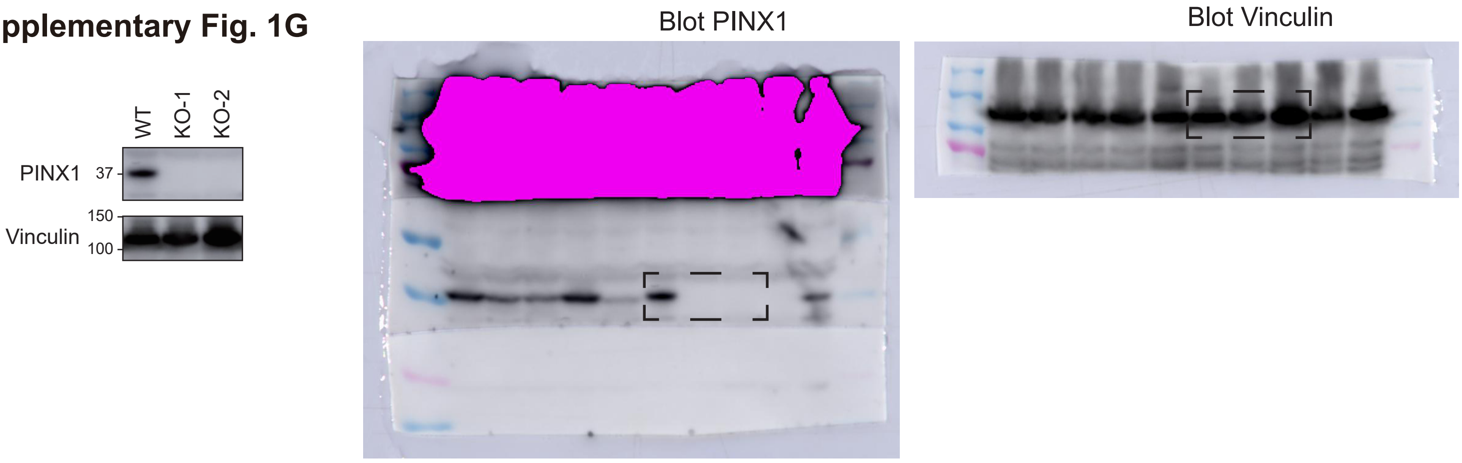

Supplementary Fig. 1H

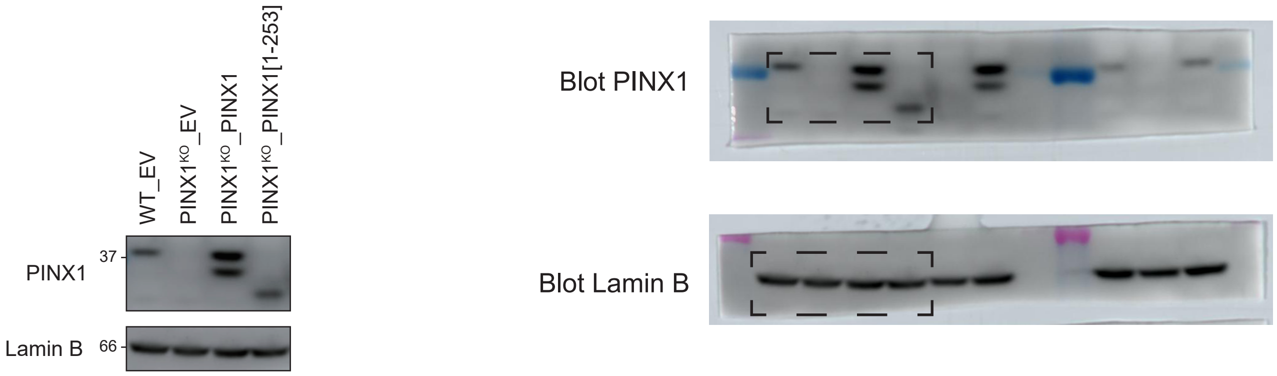

Supplementary Fig. 1I

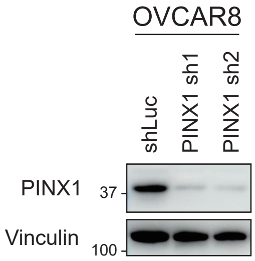

Supplementary Fig. 1K

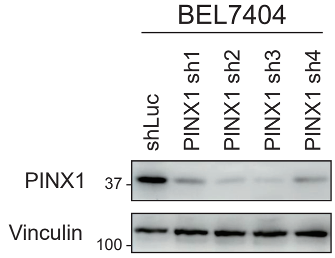

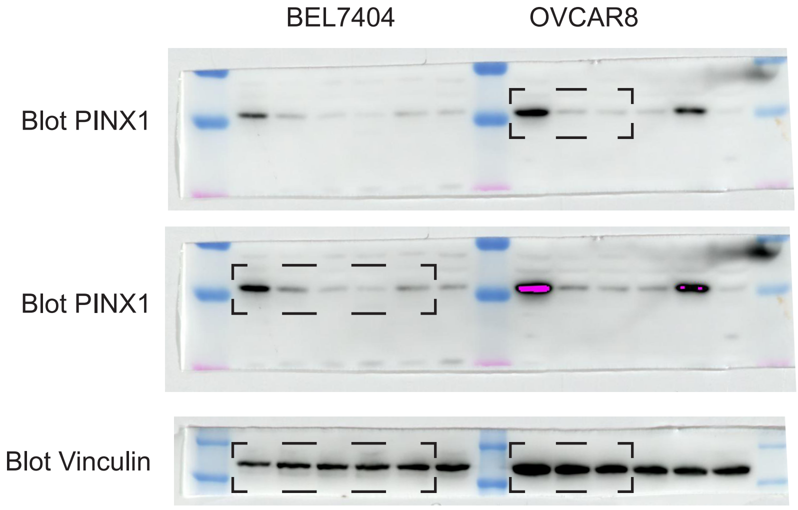

Supplementary Fig. 1J

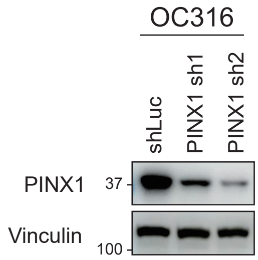

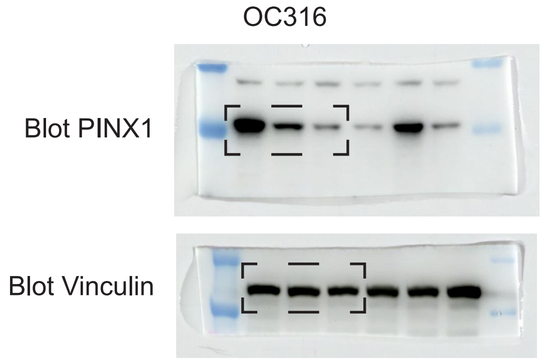

Supplementary Fig. 1L

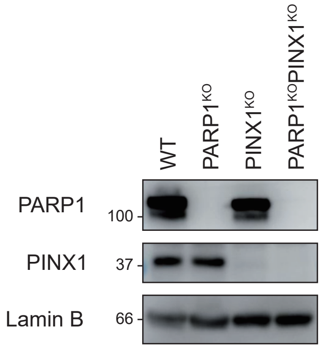

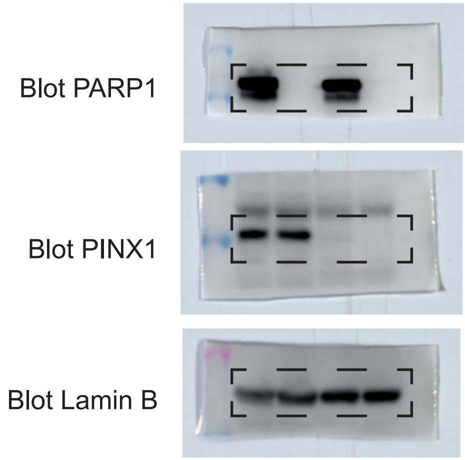

Supplementary Fig. 2A and 2J

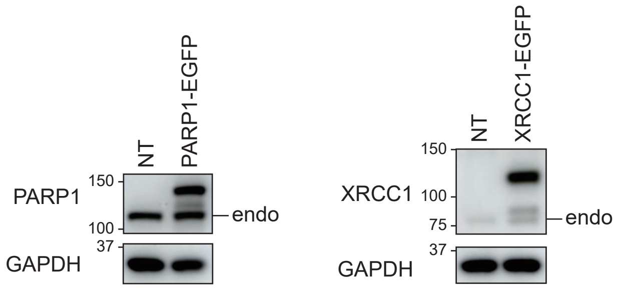

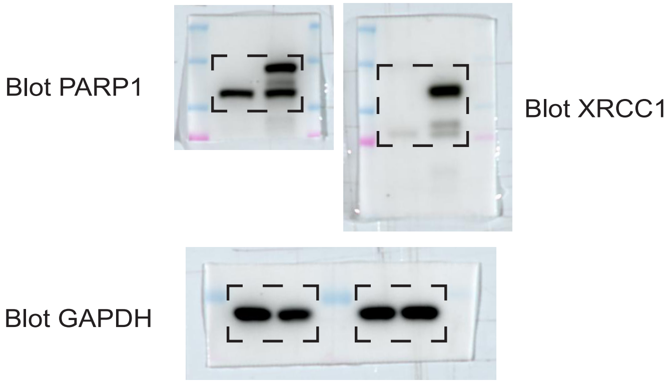

Supplementary Fig. 2B and 2K

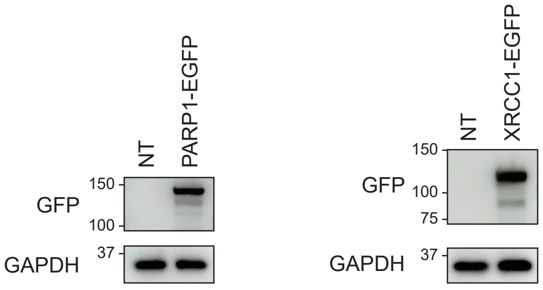

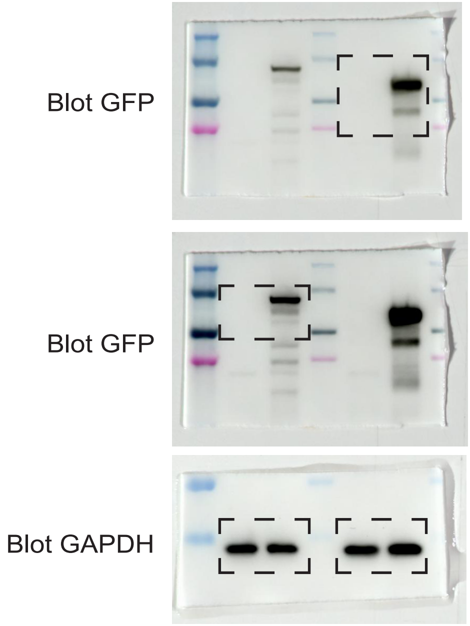

Supplementary Fig. 2C

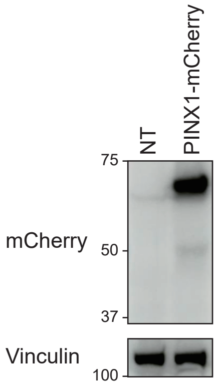

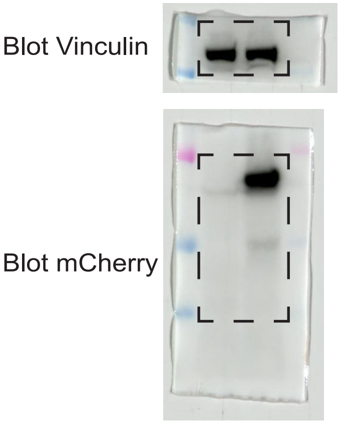

Supplementary Fig. 2E

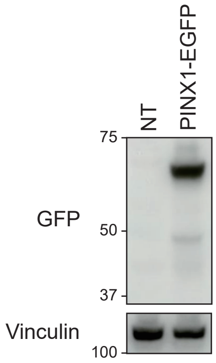

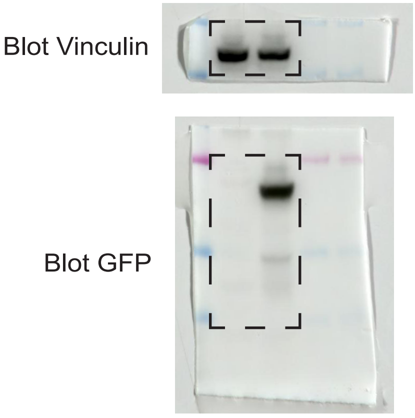

Supplementary Fig. 2D

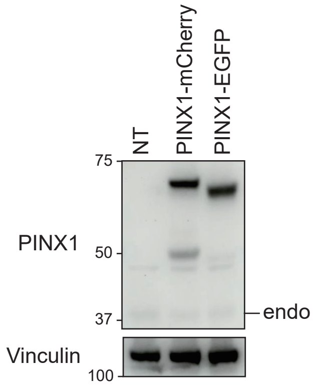

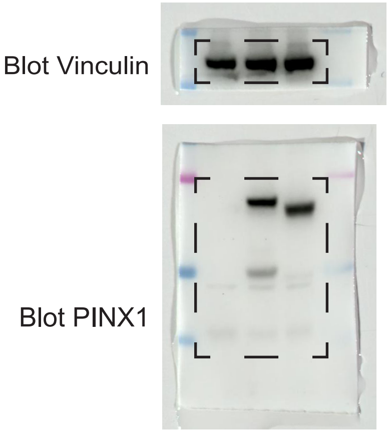

Figure 5B

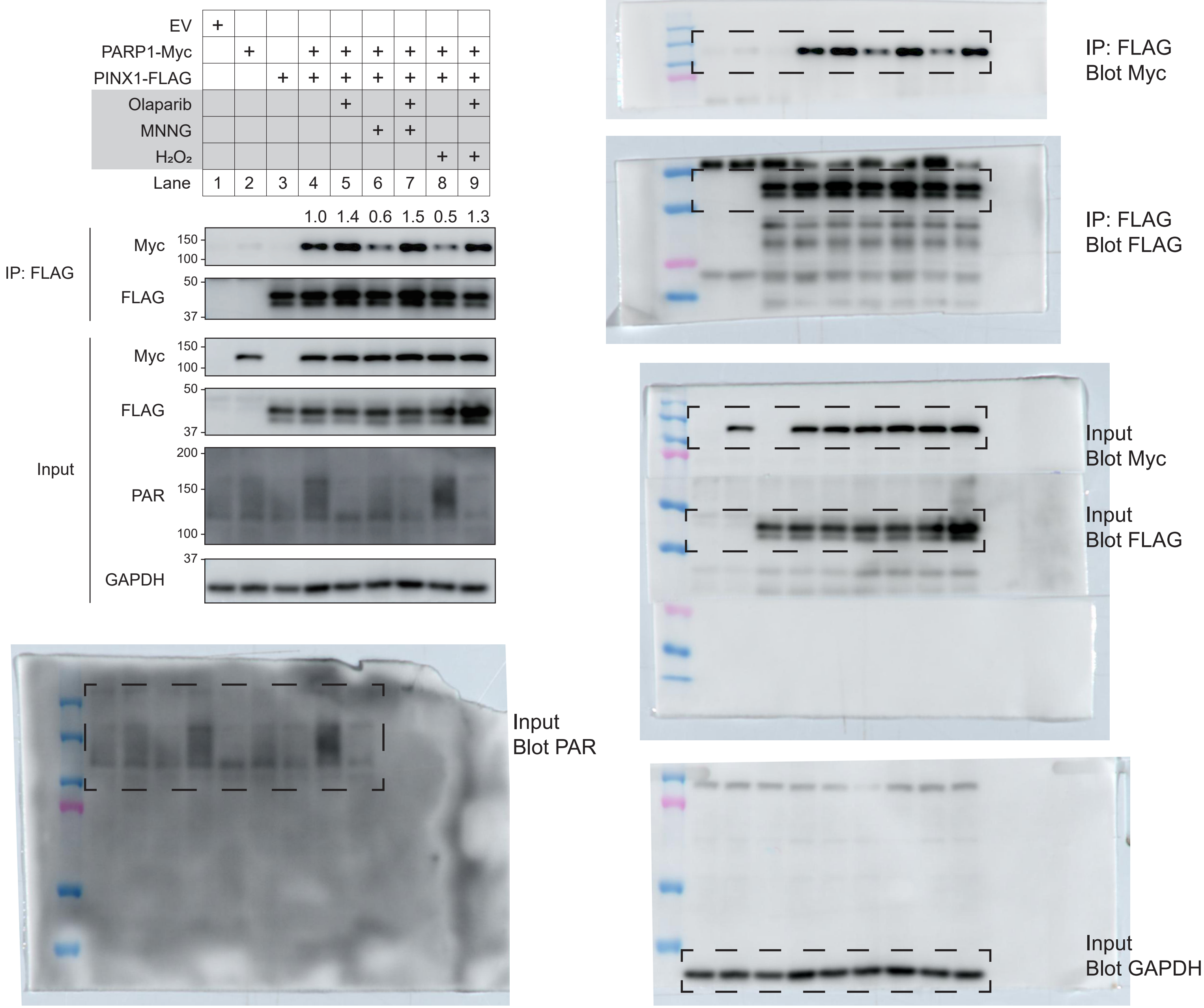

Supplementary Fig. 3A

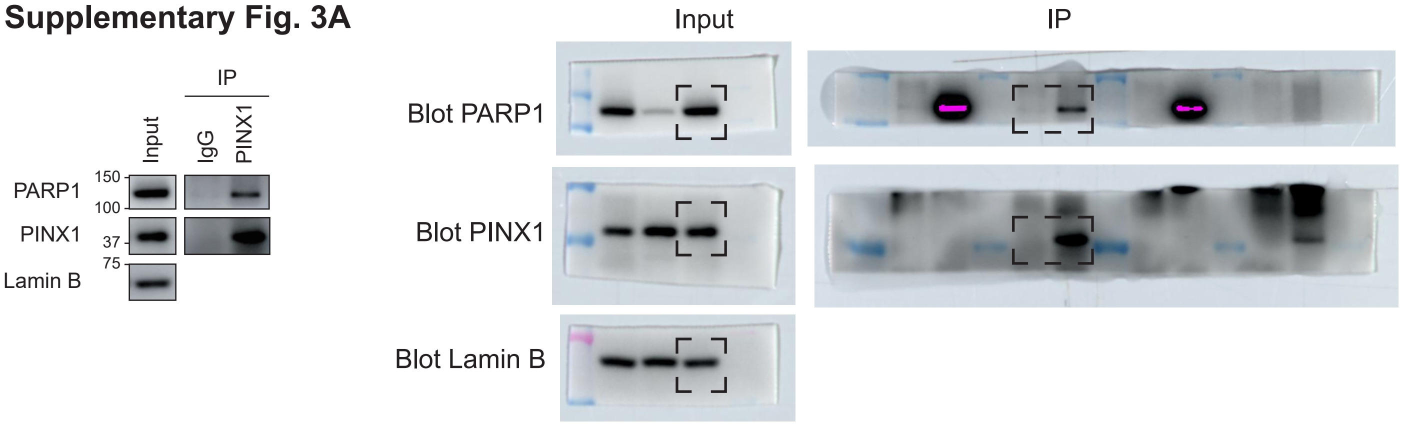

Supplementary Fig. 3B

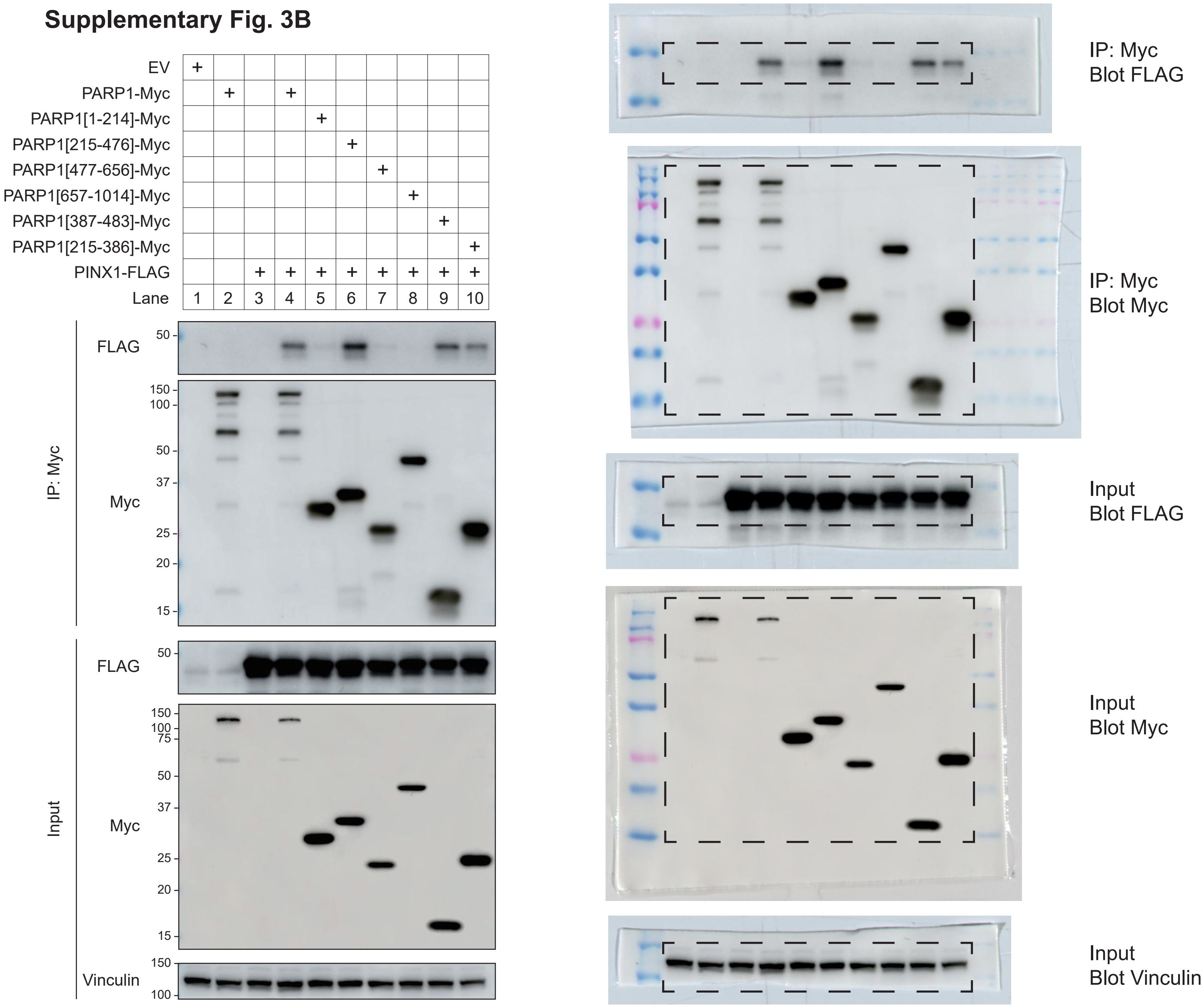

Supplementary Fig. 3D

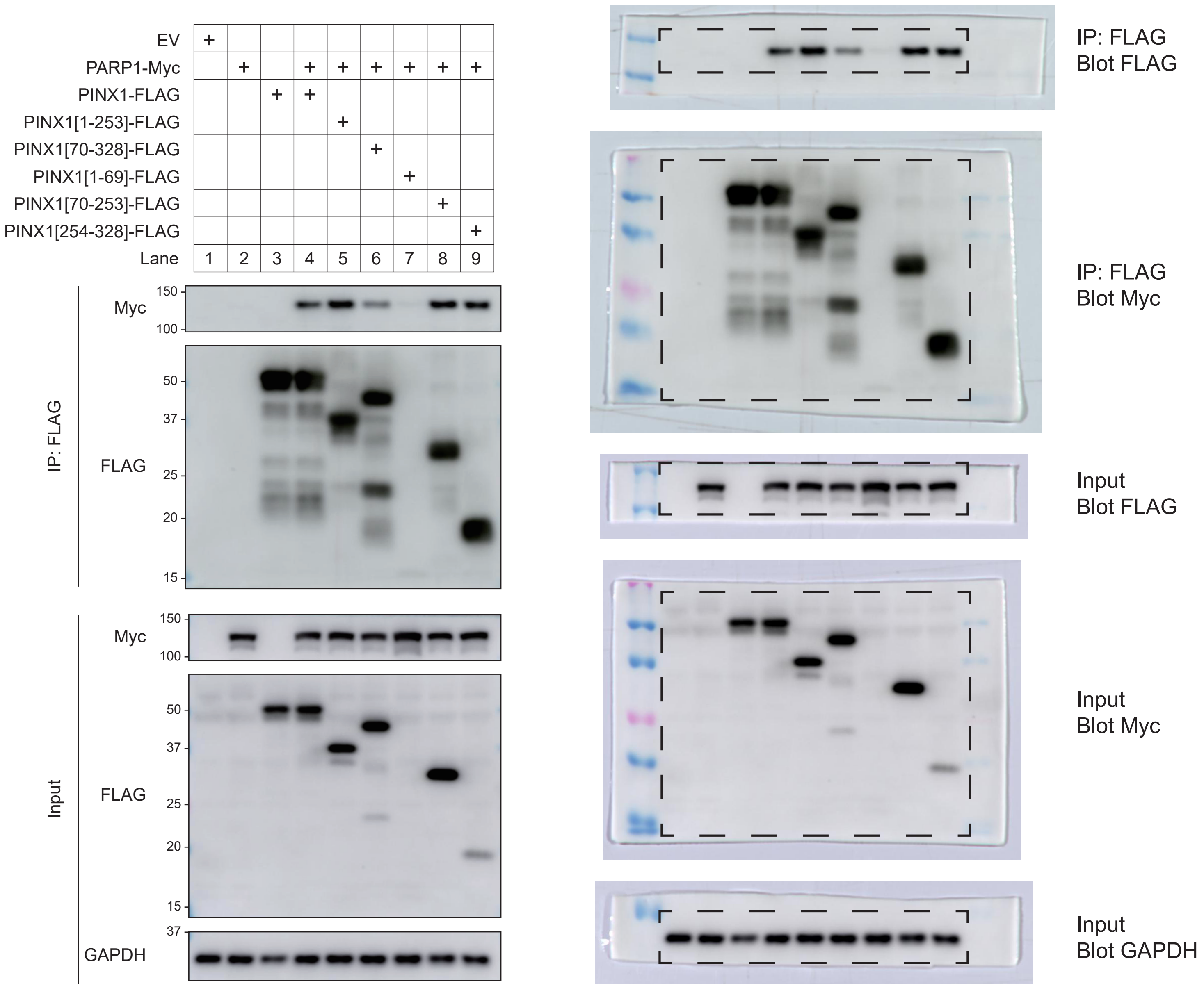

Figure 6A

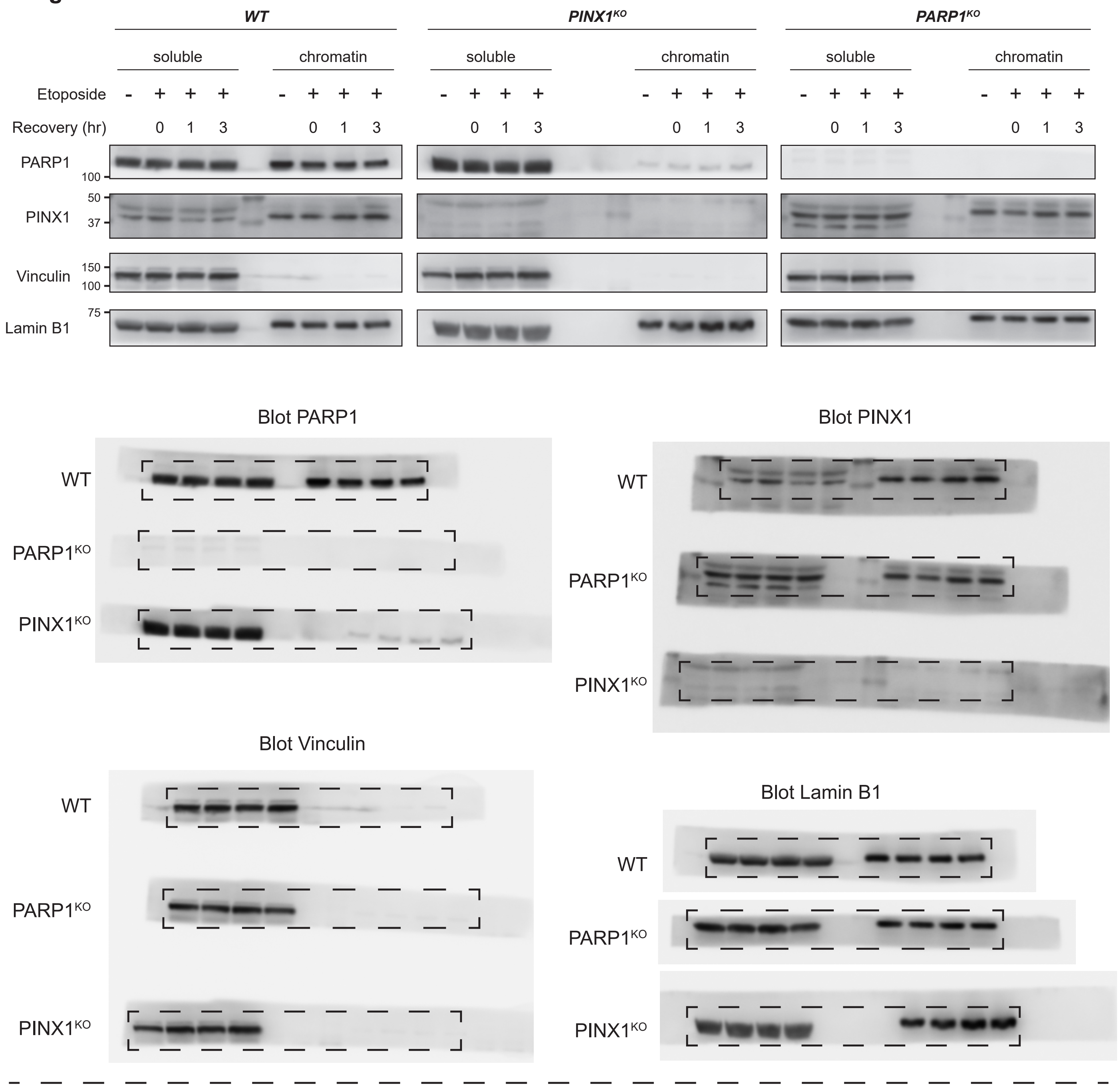
WT
PINX1KO
PARP1KO
soluble
chromatin
Etoposide - + + + - + + +
Recovery (hr) 0 1 3 0 1 3
PARP1
PINX1
Vinculin
Lamin B1
100
50
37
150
100
75
Blot PARP1
Blot PINX1
Blot Vinculin
Blot Lamin B1
WT
PARP1KO
PINX1KO

Figure 6B

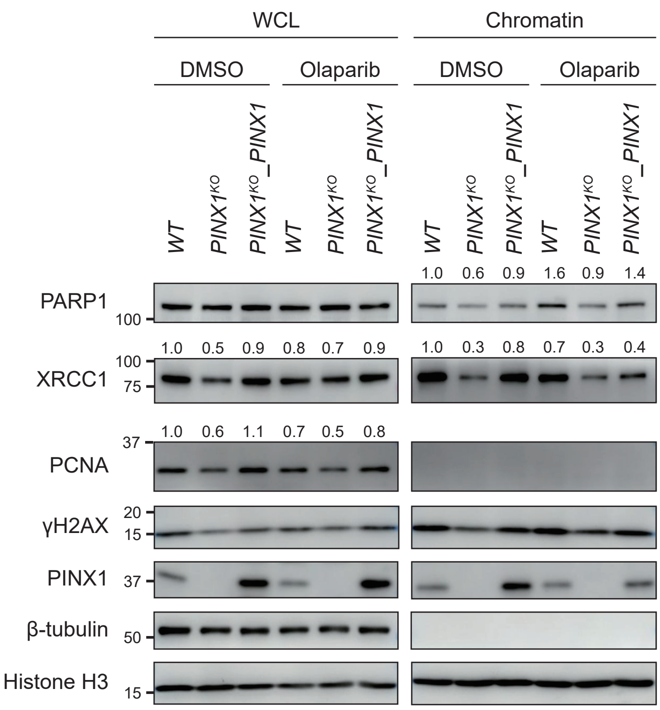
WCL
Chromatin
DMSO
Olaparib
DMSO
Olaparib
WT
PINX1KO
PINX1KO_PINX1
WT
PINX1KO
PINX1KO_PINX1
WT
PINX1KO
PINX1KO_PINX1
WT
PINX1KO
PINX1KO_PINX1
1.0 0.6 0.9 1.6 0.9 1.4
PARP1
100
1.0 0.5 0.9 0.8 0.7 0.9
1.0 0.3 0.8 0.7 0.3 0.4
100
XRCC1
75
1.0 0.6 1.1 0.7 0.5 0.8
37
PCNA
20
γH2AX
15
PINX1
37
β-tubulin
50
Histone H3
15

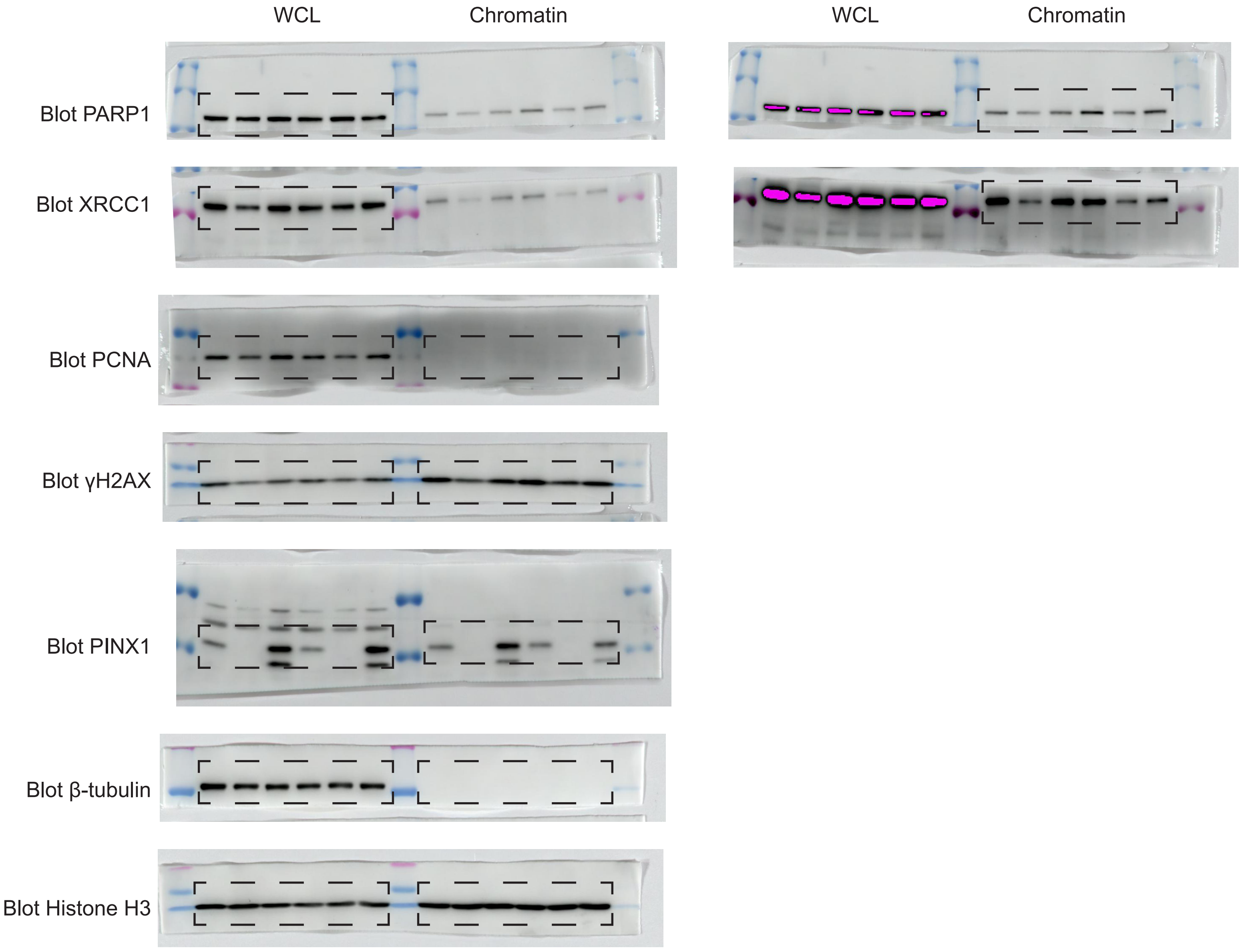
WCL
Chromatin
WCL
Chromatin
Blot PARP1
Blot XRCC1
Blot PCNA
Blot γH2AX
Blot PINX1
Blot β-tubulin
Blot Histone H3

Supplementary Fig. 4

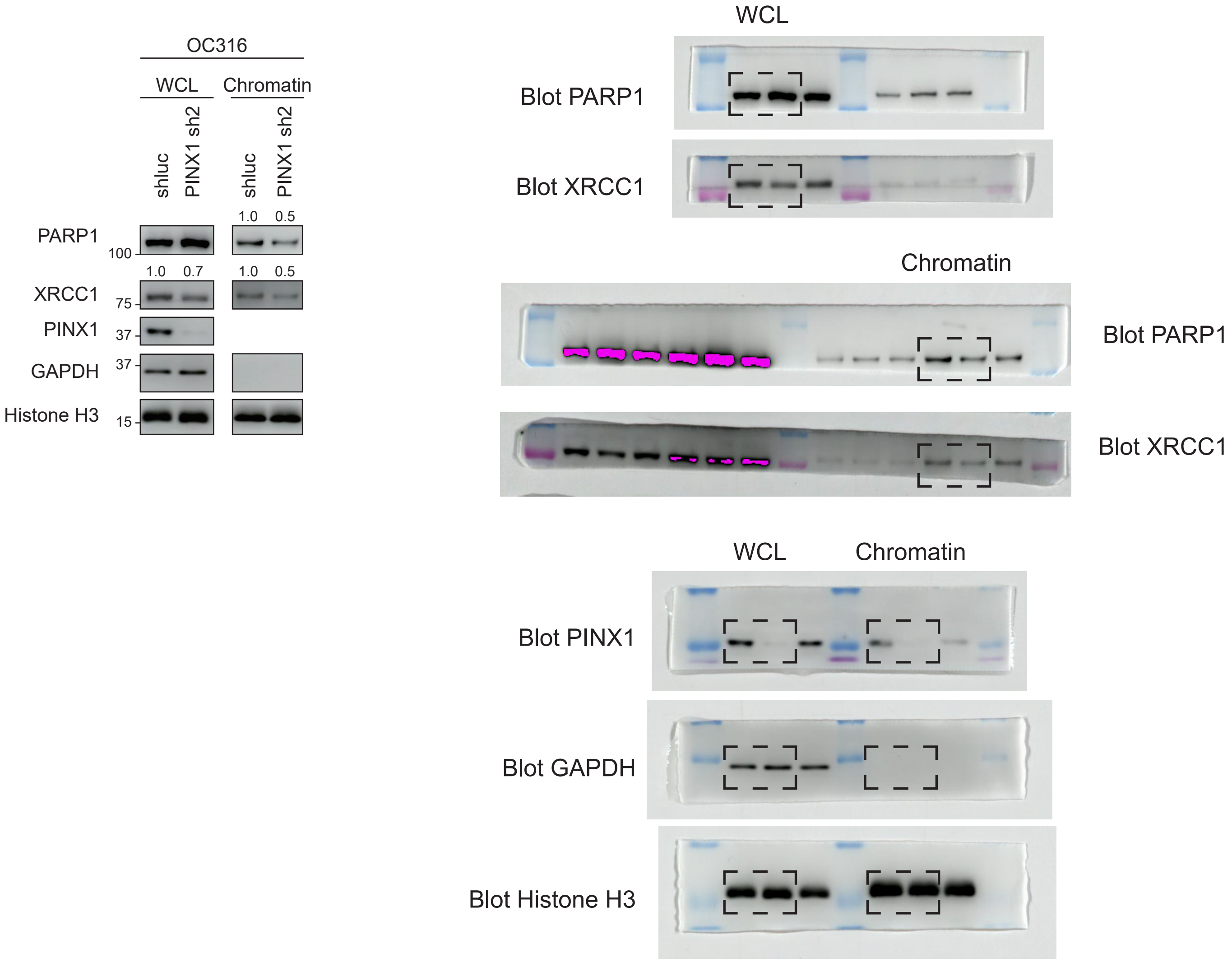

Figure 6G

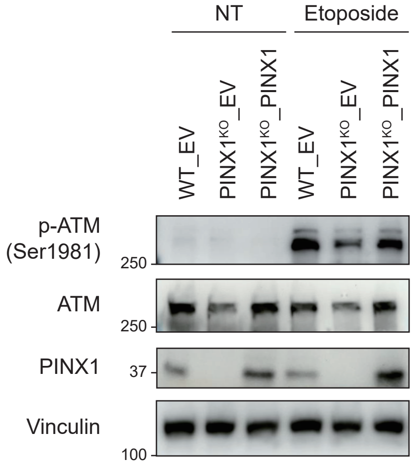

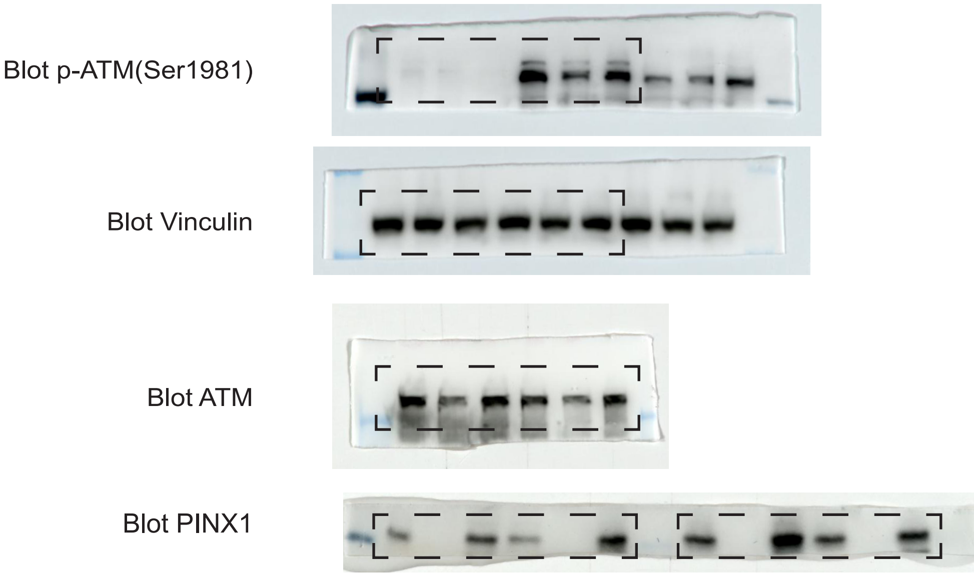

Figure 6H

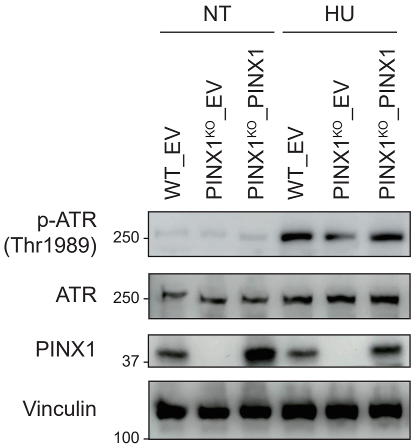

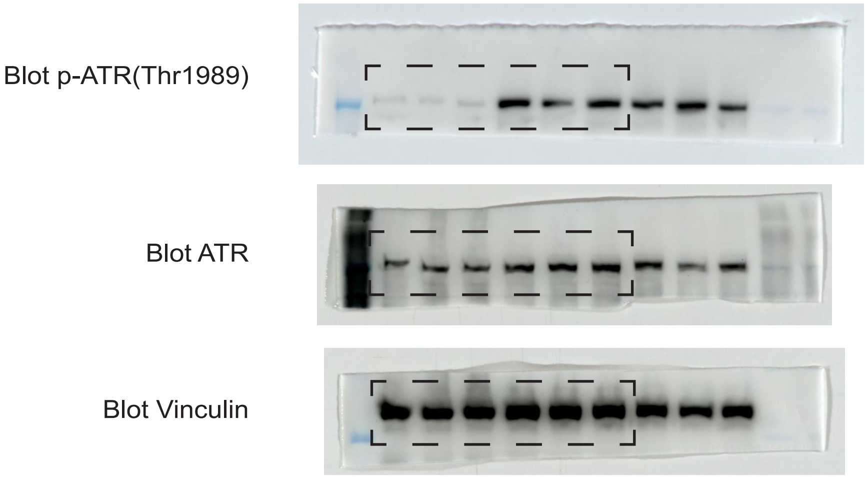

Supplement: Supplementary file 4 — uncropped western blots [file 41419_2024_7009_MOESM4_ESM.pdf]
